# Supplementary material for: The gut microbiota-immune-brain axis in a wild vertebrate: dynamic interactions and health impacts
Source: Front Microbiol. 2024 Sep 10;15:1413976. doi: 10.3389/fmicb.2024.1413976 (PMC11420037; doi:10.3389/fmicb.2024.1413976)

Supplementary Material

Table of Contents

Supplementary Material
- Table of Contents
- Table S1. Summary of final ASV table: 16s rRNA
- Table S2. Summary of final ASV table: 28s rRNA
- 1. Exploratory factor analysis for the latent variable "Immunity"
  - Table S3. Latent variable model results excluding Haptoglobin assay
  - Table S4. Latent variable model resullts incorporating Haptoglobin assay
- Figure S1. Diagrams illustrating the Bayesian structural equation models for the number of bacterial and eukaryotic ASVs.
- Figure S2. Posterior estimates (points) and 95% credible intervals (horizontal lines) from Bayesian structural equation models that include the latent variable "Immunity"
- Figure S3. Posterior estimates (points) and 95% credible intervals (horizontal lines) from Bayesian structural equation model for Haptoglobin immune assay
- Figure S4. Regression plots depicting the age effects obtained from brmsSEM: age effects on *Immunity* and f-CORT, eukaryotic Faith PD and n° of observed ASVs
- 2. Bayesian Structural Equation Modeling (SEM) diagnostics of 16S rRNA diversity measurements - models incorporating the latent variable "Immunity"
  - Table S5. Shannon diversity index
  - Table S6. Faith PD
  - Table S7. N° of observed ASV's
  - Table S8. Bayes R2 for each diversity measurement
- 3. Bayesian Structural Equation Modeling (SEM) diagnostics of 28S rRNA diversity measurements - models incorporating the latent variable "Immunity"
  - Table S9. Shannon diversity index
  - Table S10. Faith PD
  - Table S11. N° of observed ASV's
  - Table S12. Bayes R2 for each diversity measurement
- 4. Bayesian Structural Equation Modeling (SEM) diagnostics of 16S rRNA diversity measurements - model results for Haptoglobin immune assay
  - Table S13. Shannon diversity index
  - Table S14. Faith PD
  - Table S15. N° of Observed ASV's
  - Table S16. Bayes R2 for each diversity measurement
- 5. Bayesian Structural Equation Modeling (SEM) diagnostics of 28S rRNA diversity measurements - model results for Haptoglobin immune assay
  - Table S17. Shannon diversity index
  - Table S18. Faith PD
  - Table S19. N° of observed ASV's
  - Table S20. Bayes R2 for each diversity measurement
- Figure S5. Differential abundance analysis results for each of the variables in study - ANCOM-BC2 model incorporating the latent variable *Immunity*
  - Table S21. Sensitivity analysis for the two deferentially abundant taxa
- Figure S6. Differential abundance analysis results for each of the variables in study - ANCOM-BC2 model incorporating Haptoglobin immune assay
  - Table S22 Sensitivity analysis for the deferentially abundant taxa
- Figure S7. Bayesian structural equation models for the different bacterial diversity measures with results from each immune assay superimposed onto each diagram
- Figure S8. Bayesian structural equation models for the different eukaryotic diversity measures with results from each immune assay superimposed onto each diagram

Table S1. Summary of final ASV table: 16s rRNA

	Sample
**Number of samples**	86
**Number of features (ASVs)**	2,072
**Total frequency**	20,336

Table S2. Summary of final ASV table: 28s rRNA

	Sample
**Number of samples**	72
**Number of features (ASVs)**	1768
**Total frequency**	31,502

1. Exploratory factor analysis for the latent variable "Immunity"

Table S3. Latent variable model results excluding Haptoglobin assay

**Latent variables:**						
	**Estimate**	**Std.Err**	**z-value**	**P(>|z|)**	**Std.lv**	**Std.all**
immunity =~						
std_ha	0.94	0.09	10.01	0.00	0.94	0.94
std_hl	0.90	0.10	8.76	0.00	0.90	0.90
std_bka	0.31	0.12	2.58	0.01	0.31	0.31
std_lyso	0.31	0.12	2.69	0.01	0.31	0.31
std_igy	0.53	0.11	4.78	0.00	0.53	0.53
**Intercepts:**						
	**Estimate**	**Std.Err**	**z-value**	**P(>|z|)**	**Std.lv**	**Std.all**
std_ha	0.00	0.10	0.00	1.00	0.00	0.00
std_hl	0.00	0.11	0.00	1.00	0.00	0.00

std_bka	0.00	0.10	0.00	1.00	0.00	0.00
std_lyso	-0.01	0.11	-0.08	0.94	-0.01	-0.01
std_igy	0.00	0.11	0.00	1.00	0.00	0.00
immunity	0.00				0.00	0.00
**Variances:**						
	**Estimate**	**Std.Err**	**z-value**	**P(>|z|)**	**Std.lv**	**Std.all**
std_ha	0.112	0.089	1.261	0.207	0.112	0.113
std_hl	0.181	0.089	2.032	0.042	0.181	0.183
std_bka	0.895	0.164	5.456	0.000	0.895	0.906
std_lyso	0.894	0.223	4.010	0.000	0.894	0.903
std_igy	0.709	0.116	6.092	0.000	0.709	0.717
immunity	1.000				1.000	1.000
**R-Square:**	**Estimate**					
std_ha	0.887					
std_hl	0.817					
std_bka	0.094					
std_lyso	0.097					
std_igy	0.283					

Table S4. Latent variable model resullts incorporating Haptoglobin assay

**Latent Variables:**						
	**Estimate**	**Std.Err**	**z-value**	**P(>|z|)**	**Std.lv**	**Std.all**
**immunity =~**						
std_ha	0.93	0.09	10.20	0.00	0.93	0.93
std_hl	0.91	0.10	9.20	0.00	0.91	0.91
std_bka	0.31	0.11	2.77	0.01	0.31	0.31
std_lyso	0.32	0.12	2.64	0.01	0.32	0.32
std_hapto	0.26	0.15	1.76	0.08	0.26	0.26
std_igy	0.53	0.11	4.73	0.00	0.53	0.53
**Intercepts:**						
	**Estimate**	**Std.Err**	**z-value**	**P(>|z|)**	**Std.lv**	**Std.all**
.std_ha	0.00	0.10	0.00	1.00	0.00	0.00

.std_hl	0.00	0.11	0.00	1.00	0.00	0.00
.std_bka	0.00	0.10	0.00	1.00	0.00	0.00
.std_lyso	-0.01	0.11	-0.08	0.94	-0.01	-0.01
.std_hapto	-0.01	0.11	-0.09	0.93	-0.01	-0.01
.std_igy	0.00	0.11	0.00	1.00	0.00	0.00
immunity	0.00				0.00	0.00
**Variances:**						
	**Estimate**	**Std.Err**	**z-value**	**P(>|z|)**	**Std.lv**	**Std.all**
.std_ha	0.13	0.07	1.79	0.07	0.13	0.13
.std_hl	0.17	0.07	2.31	0.02	0.17	0.17
.std_bka	0.89	0.16	5.53	0.00	0.89	0.90
.std_lyso	0.89	0.22	4.09	0.00	0.89	0.90
.std_hapto	0.92	0.44	2.10	0.04	0.92	0.93
.std_igy	0.71	0.12	6.11	0.00	0.71	0.72
immunity	1.00				1.00	1.00
**R-Square:**	**Estimate**					
std_ha	0.87					
std_hl	0.83					
std_bka	0.10					
std_lyso	0.10					
std_hapto	0.07					
std_igy	0.28					

Figure S1. Diagrams illustrating the Bayesian structural equation models for the number of bacterial and eukaryotic ASVs.

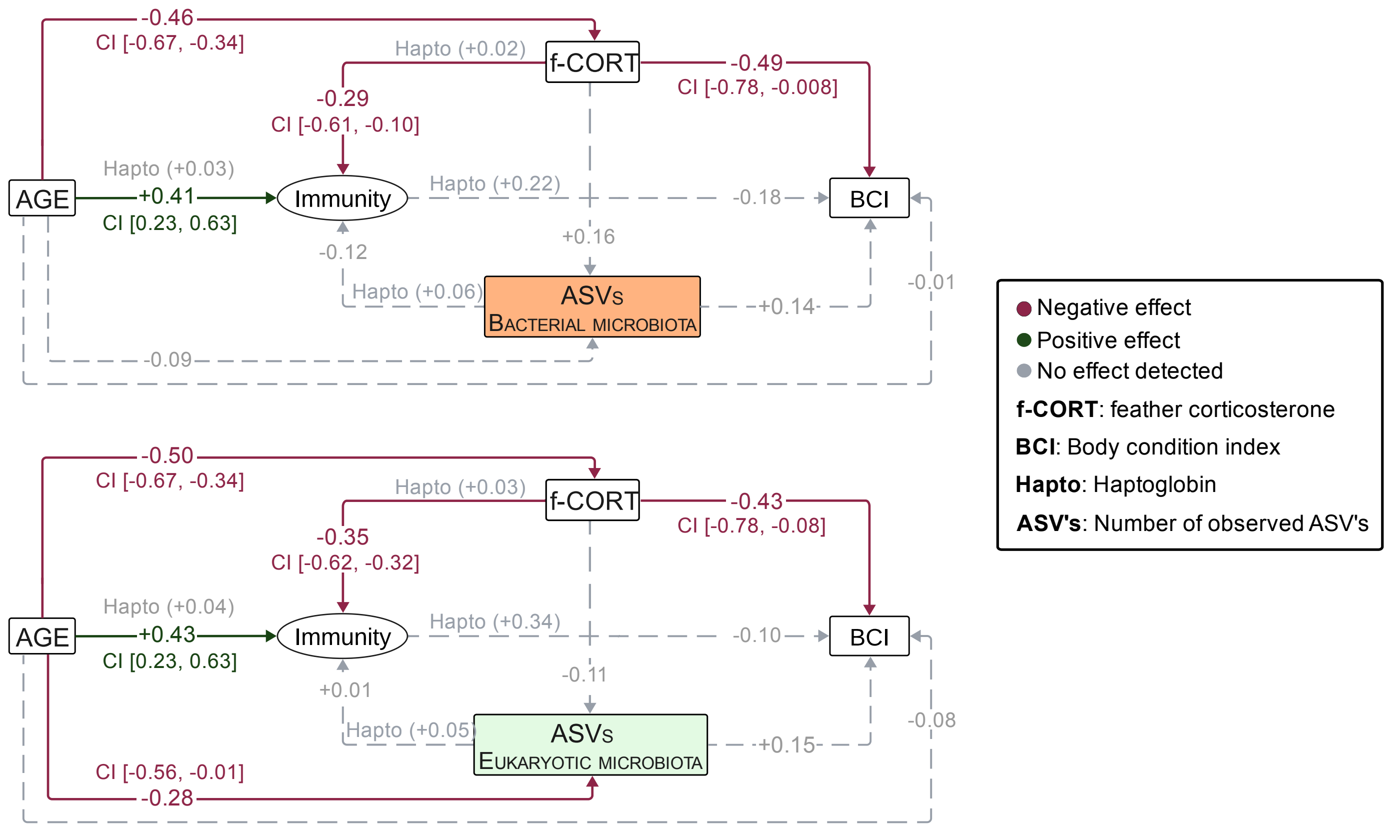

Figure S2. Posterior estimates (points) and 95% credible intervals (horizontal lines) from Bayesian structural equation models that include the latent variable "Immunity"

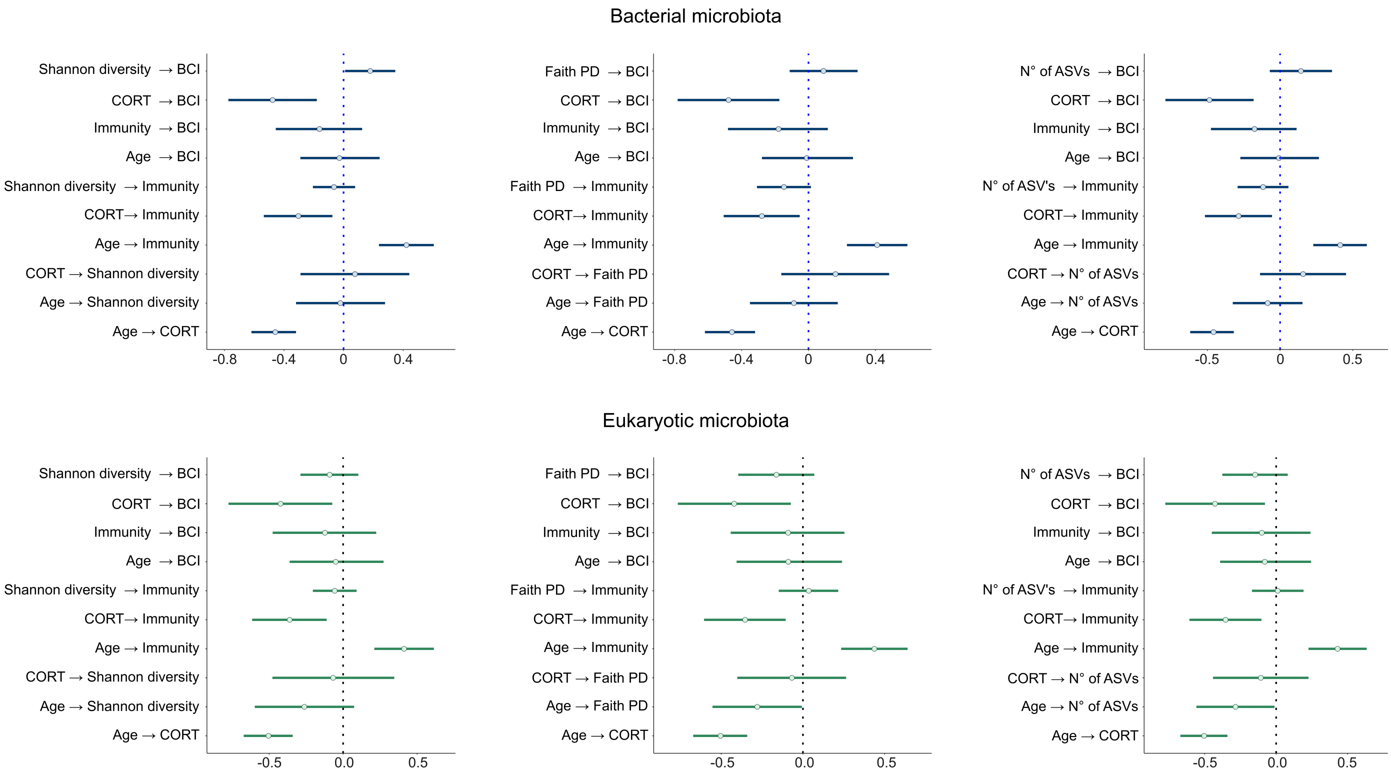

Note: A predictor confidently explains the outcome variable when the 95% credible intervals do not overlap 0.

Figure S3. Posterior estimates (points) and 95% credible intervals (horizontal lines) from Bayesian structural equation model for Haptoglobin immune assay

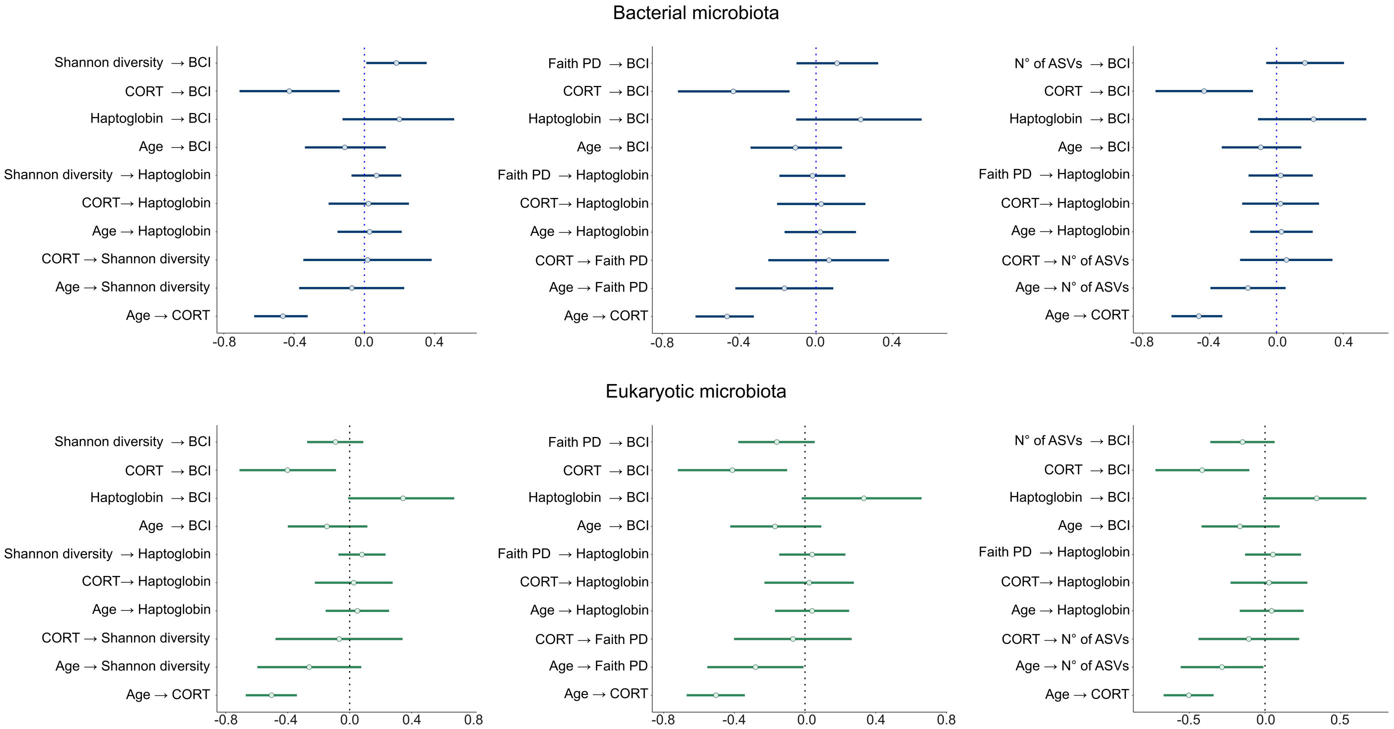

Note: A predictor confidently explains the outcome variable when the 95% credible intervals do not overlap 0.

Figure S4. Regression plots depicting the age effects obtained from brmsSEM: age effects on *Immunity* and f-CORT, eukaryotic Faith PD and n° of observed ASVs

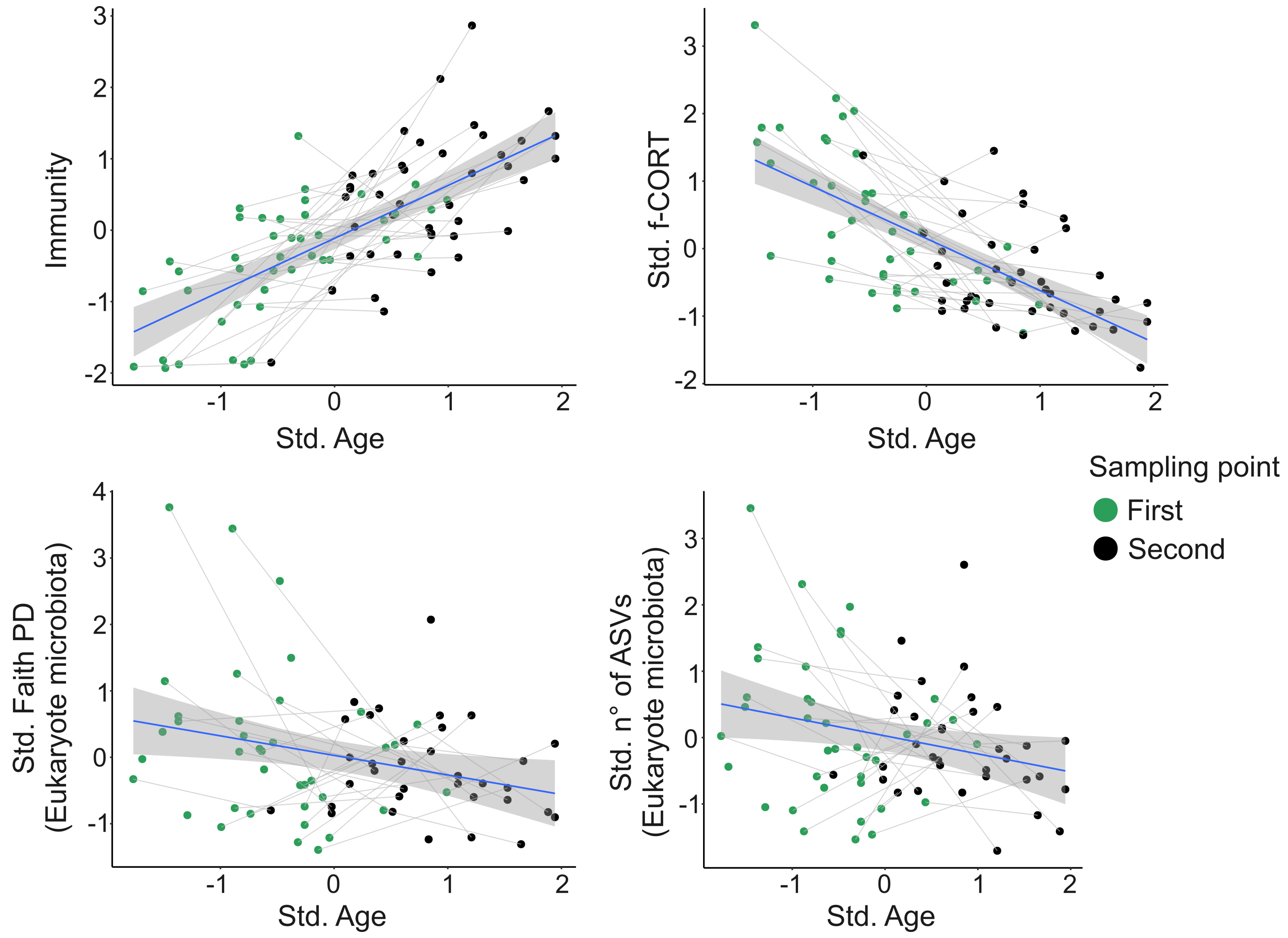

Note: Samples from the same individual at two different time points are linked by gray lines.

2. Bayesian Structural Equation Modeling (SEM) diagnostics of 16S rRNA diversity measurements - models incorporating the latent variable "Immunity"

Table S5. Shannon diversity index

**Group-Level Effects:**							
**nest (Number of levels: 23)**							
	**Estimate**	**Est.Error**	**l-95% CI**	**u-95% CI**	**Rhat**	**Bulk_ESS**	**Tail_ESS**
sd(BCI_Intercept)	0.10	0.03	0.03	0.17	1.00	40193	29967
sd(Immunity_Intercept)	0.04	0.02	0.00	0.09	1.00	53009	75934
sd(Shannon_Intercept)	0.05	0.03	0.00	0.12	1.00	73171	83559
sd(cort_Intercept)	0.04	0.03	0.00	0.10	1.00	42186	67727
**nest:ring_number (Number of levels: 43)**							
	**Estimate**	**Est.Error**	**l-95% CI**	**u-95% CI**	**Rhat**	**Bulk_ESS**	**Tail_ESS**

sd(BCI_Intercept)	0.05	0.03	0.00	0.12	1.00	34067	63400
sd(Immunity_Intercept)	0.03	0.02	0.00	0.08	1.00	55421	79441
sd(Shannon_Intercept)	0.04	0.03	0.00	0.10	1.00	85824	85327
sd(CORT_Intercept)	0.03	0.02	0.00	0.09	1.00	42011	76190
**Population-Level Effects:**							
	**Estimate**	**Est.Error**	**l-95% CI**	**u-95% CI**	**Rhat**	**Bulk_ESS**	**Tail_ESS**
BCI_Intercept	0.72	0.12	0.48	0.96	1.00	171059	152106
Immunity_Intercept	0.33	0.09	0.15	0.51	1.00	164599	148738
shannon_Intercept	0.54	0.14	0.27	0.80	1.00	152069	142448
CORT_Intercept	0.59	0.04	0.51	0.68	1.00	136837	147761
BCI_Shannon	0.18	0.09	0.01	0.35	1.00	233151	157272
BCI_CORT	-0.48	0.15	-0.77	-0.18	1.00	168613	149179
BCI_Immunity	-0.16	0.15	-0.45	0.12	1.00	135230	140900
BCI_Age	-0.03	0.14	-0.29	0.24	1.00	116247	130704
Immunity_Shannon	-0.06	0.07	-0.21	0.08	1.00	261721	154686
Immunity_CORT	-0.30	0.12	-0.54	-0.07	1.00	151185	148688
Immunity_Age	0.42	0.09	0.24	0.61	1.00	167508	151875
Shannon_CORT	0.08	0.19	-0.29	0.44	1.00	160936	152577
Shannon_Age	-0.02	0.15	-0.32	0.28	1.00	165292	153792
CORT_Age	-0.46	0.08	-0.62	-0.32	1.00	127711	150763
**Family Specific Parameters:**							
	**Estimate**	**Est.Error**	**l-95% CI**	**u-95% CI**	**Rhat**	**Bulk_ESS**	**Tail_ESS**
sigma_BCI	0.16	0.02	0.13	0.19	1.00	87455	122865
sigma_Immunity	0.14	0.01	0.11	0.17	1.00	137061	142505
sigma_Shannon	0.23	0.02	0.19	0.27	1.00	219034	145803
sigma_CORT	0.14	0.01	0.11	0.17	1.00	86466	132787
alpha_CORT	4.51	2.72	-0.57	10.31	1.00	98159	125900

Table S6. Faith PD

**Group-Level Effects:**							
**nest (Number of levels: 23)**							
	**Estimate**	**Est.Error**	**l-95% CI**	**u-95% CI**	**Rhat**	**Bulk_ESS**	**Tail_ESS**
sd(BCI_Intercept)	0.09	0.04	0.02	0.17	1.00	41835	38783

sd(Immunity_Intercept)	0.03	0.02	0.00	0.08	1.00	66785	90315
sd(FaithPD_Intercept)	0.04	0.03	0.00	0.11	1.00	78895	93112
sd(cort_Intercept)	0.04	0.03	0.00	0.10	1.00	46183	76222
**nest:ring_number (Number of levels: 43)**							
	**Estimate**	**Est.Error**	**l-95% CI**	**u-95% CI**	**Rhat**	**Bulk_ESS**	**Tail_ESS**
sd(BCI_Intercept)	0.05	0.03	0.00	0.13	1.00	36858	69470
sd(Immunity_Intercept)	0.03	0.02	0.00	0.08	1.00	72728	93078
sd(FaithPD_Intercept)	0.04	0.03	0.00	0.10	1.00	86094	94672
sd(CORT_Intercept)	0.03	0.02	0.00	0.09	1.00	45689	86842
**Population-Level Effects:**							
	**Estimate**	**Est.Error**	**l-95% CI**	**u-95% CI**	**Rhat**	**Bulk_ESS**	**Tail_ESS**
BCI_Intercept	0.78	0.13	0.53	1.03	1.00	227253	158046
Immunity_Intercept	0.35	0.09	0.18	0.53	1.00	217297	152915
FaithPD_Intercept	0.41	0.12	0.18	0.65	1.00	201266	150828
CORT_Intercept	0.59	0.04	0.51	0.68	1.00	163851	158106
BCI_FaithPD	0.09	0.10	-0.11	0.29	1.00	241820	154659
BCI_CORT	-0.48	0.16	-0.78	-0.17	1.00	221946	161268
BCI_Immunity	-0.18	0.15	-0.48	0.12	1.00	176801	155537
BCI_Age	-0.01	0.14	-0.28	0.27	1.00	148433	151120
Immunity_FaithPD	-0.15	0.08	-0.31	0.01	1.00	281668	150324
Immunity_CORT	-0.28	0.12	-0.51	-0.05	1.00	192357	158346
Immunity_Age	0.41	0.09	0.23	0.59	1.00	214708	162819
FaithPD_CORT	0.16	0.16	-0.16	0.48	1.00	204099	161576
FaithPD_Age	-0.09	0.13	-0.35	0.18	1.00	212116	161601
CORT_Age	-0.46	0.08	-0.62	-0.32	1.00	153,019.85	159,412.54
**Family Specific Parameters:**							
	**Estimate**	**Est.Error**	**l-95% CI**	**u-95% CI**	**Rhat**	**Bulk_ESS**	**Tail_ESS**
sigma_BCI	0.16	0.02	0.13	0.20	1.00	92480	124753
sigma_Immunity	0.14	0.01	0.12	0.16	1.00	195882	149848
sigma_FaithPD	0.20	0.02	0.17	0.24	1.00	241036	152012
sigma_CORT	0.14	0.01	0.11	0.17	1.00	97127	139061
alpha_CORT	4.53	2.72	-0.56	10.33	1.00	114529	138100

Table S7. N° of observed ASV's

**Group-Level Effects:**							
**nest (Number of levels: 23)**							
	**Estimate**	**Est.Error**	**l-95% CI**	**u-95% CI**	**Rhat**	**Bulk_ESS**	**Tail_ESS**
sd(BCI_Intercept)	0.09	0.04	0.02	0.17	1.00	42963	38463
sd(Immunity_Intercept)	0.03	0.02	0.00	0.09	1.00	63091	90077
sd(ASV_Intercept)	0.04	0.03	0.00	0.10	1.00	81158	94442
sd(cort_Intercept)	0.04	0.03	0.00	0.10	1.00	46623	73961
**nest:ring_number (Number of levels: 43)**							
	**Estimate**	**Est.Error**	**l-95% CI**	**u-95% CI**	**Rhat**	**Bulk_ESS**	**Tail_ESS**
sd(BCI_Intercept)	0.05	0.03	0.00	0.13	1.00	38749	74160
sd(Immunity_Intercept)	0.03	0.02	0.00	0.08	1.00	68082	90282
sd(ASV_Intercept)	0.03	0.02	0.00	0.08	1.00	101607	98436
sd(CORT_Intercept)	0.03	0.02	0.00	0.09	1.00	45851	87339
**Population-Level Effects:**							
	**Estimate**	**Est.Error**	**l-95% CI**	**u-95% CI**	**Rhat**	**Bulk_ESS**	**Tail_ESS**
BCI_Intercept	0.77	0.12	0.52	1.01	1.00	221975	158089
Immunity_Intercept	0.33	0.09	0.16	0.51	1.00	209396	146766
ASV_Intercept	0.33	0.11	0.12	0.55	1.00	204922	146073
CORT_Intercept	0.59	0.04	0.51	0.68	1.00	164348	158239
BCI_ASV	0.14	0.11	-0.07	0.36	1.00	278133	153518
BCI_CORT	-0.49	0.15	-0.79	-0.18	1.00	216771	153897
BCI_Immunity	-0.18	0.15	-0.48	0.11	1.00	175698	144150
BCI_Age	-0.01	0.14	-0.27	0.27	1.00	149260	143820
Immunity_ASV	-0.12	0.09	-0.29	0.06	1.00	273104	155751
Immunity_CORT	-0.29	0.12	-0.52	-0.06	1.00	187571	151720
Immunity_Age	0.41	0.09	0.23	0.60	1.00	213409	156429
ASV_CORT	0.16	0.15	-0.14	0.45	1.00	209768	159805
ASV_Age	-0.09	0.12	-0.33	0.15	1.00	213539	159926
CORT_Age	-0.46	0.08	-0.62	-0.32	1.00	153667	162653
**Family Specific Parameters:**							
	**Estimate**	**Est.Error**	**l-95% CI**	**u-95% CI**	**Rhat**	**Bulk_ESS**	**Tail_ESS**
sigma_BCI	0.16	0.02	0.13	0.20	1.00	94855	130217
sigma_Immunity	0.14	0.01	0.12	0.16	1.00	183033	152022
sigma_ASV	0.19	0.02	0.16	0.22	1.00	261966	150278

sigma_CORT	0.14	0.01	0.11	0.17	1.00	101905	141945
alpha_CORT	4.52	2.72	-0.58	10.33	1.00	113172	139327

Table S8. Bayes R2 for each diversity measurement

	**R2m**	**Est.Error**	**Q2.5**	**Q97.5**	**R2c**	**Est.Error**	**Q2.5**	**Q97.5**
**BCI (Path1)**	0.19	0.07	0.07	0.32	0.47	0.08	0.28	0.61
**Immunity (Path2)**	0.53	0.05	0.41	0.61	0.57	0.06	0.44	0.67
**Shanonn (Path3)**	0.03	0.03	0.00	0.11	0.10	0.06	0.01	0.25
**CORT (Path4)**	0.35	0.08	0.19	0.51	0.46	0.11	0.24	0.65
	**R2m**	**Est.Error**	**Q2.5**	**Q97.5**	**R2c**	**Est.Error**	**Q2.5**	**Q97.5**
**BCI (Path1)**	0.17	0.07	0.05	0.30	0.44	0.09	0.24	0.59
**Immunity (Path2)**	0.54	0.05	0.42	0.63	0.57	0.05	0.45	0.67
**Faith PD (Path3)**	0.08	0.05	0.00	0.19	0.15	0.07	0.03	0.31
**CORT (Path4)**	0.35	0.08	0.19	0.51	0.46	0.11	0.24	0.65
	**R2m**	**Est.Error**	**Q2.5**	**Q97.5**	**R2c**	**Est.Error**	**Q2.5**	**Q97.5**
**BCI (Path1)**	0.18	0.07	0.06	0.31	0.45	0.09	0.25	0.60
**Immunity (Path2)**	0.53	0.05	0.41	0.62	0.57	0.06	0.44	0.67
**ASV (Path3)**	0.08	0.05	0.01	0.20	0.15	0.07	0.03	0.29
**CORT (Path4)**	0.35	0.08	0.19	0.51	0.46	0.11	0.24	0.65

3. Bayesian Structural Equation Modeling (SEM) diagnostics of 28S rRNA diversity measurements - models incorporating the latent variable "Immunity"

Table S9. Shannon diversity index

**Group-Level Effects:**							
**nest (Number of levels: 23)**							
	**Estimate**	**Est.Error**	**l-95% CI**	**u-95% CI**	**Rhat**	**Bulk_ESS**	**Tail_ESS**
sd(BCI_Intercept)	0.10	0.04	0.01	0.18	1.00	43440	47029
sd(Immunity_Intercept)	0.05	0.03	0.00	0.12	1.00	46497	80602

sd(Shannon_Intercept)	0.05	0.04	0.00	0.13	1.00	88109	100503
sd(CORT_Intercept)	0.05	0.03	0.00	0.12	1.00	44588	73408
**nest:ring_number (Number of levels: 41)**							
	**Estimate**	**Est.Error**	**l-95% CI**	**u-95% CI**	**Rhat**	**Bulk_ESS**	**Tail_ESS**
sd(BCI_Intercept)	0.05	0.04	0.00	0.13	1.00	51191	82715
sd(Immunity_Intercept)	0.04	0.03	0.00	0.09	1.00	57078	97148
sd(Shannon_Intercept)	0.05	0.04	0.00	0.13	1.00	86785	101805
sd(CORT_Intercept)	0.04	0.03	0.00	0.09	1.00	52082	94318
**Population-Level Effects:**							
	**Estimate**	**Est.Error**	**l-95% CI**	**u-95% CI**	**Rhat**	**Bulk_ESS**	**Tail_ESS**
BCI_Intercept	0.84	0.16	0.53	1.15	1.00	257492	157983
Immunity_Intercept	0.35	0.11	0.14	0.56	1.00	210031	153012
shannon_Intercept	0.77	0.15	0.47	1.07	1.00	234697	151070
CORT_Intercept	0.62	0.05	0.53	0.72	1.00	235559	171146
BCI_Shannon	-0.09	0.10	-0.29	0.10	1.00	299121	151667
BCI_CORT	-0.42	0.18	-0.77	-0.07	1.00	241261	161921
BCI_Immunity	-0.12	0.18	-0.48	0.22	1.00	177201	151056
BCI_Age	-0.05	0.16	-0.36	0.27	1.00	159380	146270
Immunity_Shannon	-0.06	0.07	-0.20	0.09	1.00	323971	160165
Immunity_CORT	-0.36	0.13	-0.61	-0.11	1.00	183336	158445
Immunity_Age	0.41	0.10	0.21	0.61	1.00	226141	162064
Shannon_CORT	-0.07	0.21	-0.48	0.35	1.00	227697	159630
Shannon_Age	-0.26	0.17	-0.60	0.07	1.00	237093	162077
CORT_Age	-0.50	0.08	-0.67	-0.34	1.00	205533	170103
**Family Specific Parameters:**							
	**Estimate**	**Est.Error**	**l-95% CI**	**u-95% CI**	**Rhat**	**Bulk_ESS**	**Tail_ESS**
sigma_BCI	0.17	0.02	0.13	0.21	1.00	94141	129645
sigma_Immunity	0.13	0.01	0.11	0.16	1.00	125995	148081
sigma_Shannon	0.24	0.02	0.19	0.28	1.00	250653	151912
sigma_CORT	0.14	0.02	0.11	0.17	1.00	115629	143287
alpha_CORT	3.11	2.71	-1.78	8.93	1.00	105352	121969

Table S10. Faith PD

**Group-Level Effects:**							
**nest (Number of levels: 23)**							
	**Estimate**	**Est.Error**	**l-95% CI**	**u-95% CI**	**Rhat**	**Bulk_ESS**	**Tail_ESS**
sd(BCI_Intercept)	0.10	0.04	0.01	0.18	1.00	44108	50748
sd(Immunity_Intercept)	0.05	0.03	0.00	0.11	1.00	48959	85818
sd(FaithPD_Intercept)	0.03	0.03	0.00	0.10	1.00	107439	111926
sd(CORT_Intercept)	0.05	0.03	0.00	0.12	1.00	47224	78299
**nest:ring_number (Number of levels: 41)**							
	**Estimate**	**Est.Error**	**l-95% CI**	**u-95% CI**	**Rhat**	**Bulk_ESS**	**Tail_ESS**
sd(BCI_Intercept)	0.05	0.03	0.00	0.13	1.00	54540	88979
sd(Immunity_Intercept)	0.04	0.03	0.00	0.10	1.00	56684	99592
sd(FaithPD_Intercept)	0.04	0.03	0.00	0.10	1.00	104537	108735
sd(CORT_Intercept)	0.04	0.03	0.00	0.09	1.00	55175	95121
**Population-Level Effects:**							
	**Estimate**	**Est.Error**	**l-95% CI**	**u-95% CI**	**Rhat**	**Bulk_ESS**	**Tail_ESS**
BCI_Intercept	0.84	0.14	0.56	1.11	1.00	278684	160729
Immunity_Intercept	0.29	0.10	0.09	0.49	1.00	243151	154233
FaithPD_Intercept	0.45	0.13	0.20	0.69	1.00	271811	154780
CORT_Intercept	0.62	0.05	0.53	0.72	1.00	250796	171645
BCI_FaithPD	-0.16	0.12	-0.40	0.07	1.00	340822	161568
BCI_CORT	-0.42	0.18	-0.77	-0.07	1.00	273914	160657
BCI_Immunity	-0.09	0.18	-0.44	0.25	1.00	192620	149586
BCI_Age	-0.09	0.16	-0.41	0.24	1.00	165692	146731
Immunity_FaithPD	0.04	0.09	-0.15	0.22	1.00	341797	155995
Immunity_CORT	-0.35	0.13	-0.60	-0.11	1.00	204113	163518
Immunity_Age	0.44	0.10	0.23	0.64	1.00	251992	163363
FaithPD_CORT	-0.07	0.17	-0.40	0.26	1.00	264045	164815
FaithPD_Age	-0.28	0.14	-0.55	-0.01	1.00	264698	163463
CORT_Age	-0.50	0.08	-0.67	-0.34	1.00	217734	170138
**Family Specific Parameters:**							
	**Estimate**	**Est.Error**	**l-95% CI**	**u-95% CI**	**Rhat**	**Bulk_ESS**	**Tail_ESS**
sigma_BCI	0.17	0.02	0.13	0.21	1.00	98802	132296
sigma_Immunity	0.13	0.01	0.11	0.16	1.00	122836	149890
sigma_FaithPD	0.19	0.02	0.16	0.23	1.00	316900	148549

sigma_CORT	0.14	0.02	0.11	0.17	1.00	122290	142987
alpha_CORT	3.11	2.72	-1.78	8.96	1.00	114427	131169

Table S11. N° of observed ASV's

**Group-Level Effects:**							
**nest (Number of levels: 23)**							
	**Estimate**	**Est.Error**	**l-95% CI**	**u-95% CI**	**Rhat**	**Bulk_ESS**	**Tail_ESS**
sd(BCI_Intercept)	0.10	0.04	0.01	0.18	1.00	41708	43096
sd(Immunity_Intercept)	0.05	0.03	0.00	0.11	1.00	46701	75331
sd(ASV_Intercept)	0.03	0.02	0.00	0.09	1.00	111097	94406
sd(CORT_Intercept)	0.05	0.03	0.00	0.12	1.00	42664	69630
**nest:ring_number (Number of levels: 41)**							
	**Estimate**	**Est.Error**	**l-95% CI**	**u-95% CI**	**Rhat**	**Bulk_ESS**	**Tail_ESS**
sd(BCI_Intercept)	0.05	0.03	0.00	0.13	1.00	49548	82494
sd(Immunity_Intercept)	0.04	0.03	0.00	0.10	1.00	52291	87013
sd(ASV_Intercept)	0.04	0.03	0.00	0.11	1.00	85524	92005
sd(CORT_Intercept)	0.04	0.03	0.00	0.09	1.00	46236	90325
**Population-Level Effects:**							
	**Estimate**	**Est.Error**	**l-95% CI**	**u-95% CI**	**Rhat**	**Bulk_ESS**	**Tail_ESS**
BCI_Intercept	0.84	0.14	0.56	1.13	1.00	193481	152637
Immunity_Intercept	0.30	0.10	0.09	0.50	1.00	169514	144525
ASV_Intercept	0.52	0.13	0.27	0.77	1.00	182509	140896
CORT_Intercept	0.62	0.05	0.53	0.72	1.00	186895	161248
BCI_ASV	-0.15	0.12	-0.38	0.08	1.00	254699	156980
BCI_CORT	-0.43	0.18	-0.78	-0.08	1.00	189445	155658
BCI_Immunity	-0.10	0.18	-0.45	0.24	1.00	159502	150111
BCI_Age	-0.08	0.16	-0.39	0.24	1.00	136058	137626
Immunity_ASV	0.01	0.09	-0.17	0.19	1.00	245230	156711
Immunity_CORT	-0.35	0.13	-0.61	-0.10	1.00	157148	154855
Immunity_Age	0.43	0.10	0.23	0.63	1.00	185126	157534
ASV_CORT	-0.11	0.17	-0.44	0.23	1.00	189017	151191
ASV_Age	-0.28	0.14	-0.56	-0.01	1.00	190401	153832
CORT_Age	-0.50	0.08	-0.67	-0.34	1.00	168839	164270

**Family Specific Parameters:**							
	**Estimate**	**Est.Error**	**l-95% CI**	**u-95% CI**	**Rhat**	**Bulk_ESS**	**Tail_ESS**
sigma_BCI	0.17	0.02	0.13	0.21	1.00	89208	125835
sigma_Immunity	0.13	0.01	0.11	0.16	1.00	110137	141585
sigma_ASV	0.19	0.02	0.16	0.23	1.00	215140	149817
sigma_CORT	0.14	0.02	0.11	0.17	1.00	105791	134827
alpha_CORT	3.13	2.72	-1.73	9.00	1.00	92657	111289

Table S12. Bayes R2 for each diversity measurement

	**R2m**	**Est.Error**	**Q2.5**	**Q97.5**	**R2c**	**Est.Error**	**Q2.5**	**Q97.5**
**BCI (Path1)**	0.16	0.07	0.04	0.30	0.41	0.10	0.19	0.58
**Immunity (Path2)**	0.58	0.05	0.46	0.66	0.64	0.06	0.50	0.74
**Shannon (Path3)**	0.08	0.05	0.00	0.20	0.16	0.08	0.03	0.33
**CORT (Path4)**	0.41	0.09	0.22	0.56	0.54	0.10	0.30	0.70
	**R2m**	**Est.Error**	**Q2.5**	**Q97.5**	**R2c**	**Est.Error**	**Q2.5**	**Q97.5**
**BCI (Path1)**	0.17	0.07	0.04	0.30	0.42	0.10	0.20	0.59
**Immunity (Path2)**	0.58	0.05	0.45	0.66	0.64	0.06	0.50	0.74
**Faith PD (Path3)**	0.11	0.06	0.01	0.25	0.17	0.07	0.04	0.33
**CORT (Path4)**	0.41	0.09	0.22	0.56	0.54	0.10	0.30	0.70
	**R2m**	**Est.Error**	**Q2.5**	**Q97.5**	**R2c**	**Est.Error**	**Q2.5**	**Q97.5**
**BCI (Path1)**	0.16	0.07	0.04	0.30	0.42	0.10	0.20	0.59
**Immunity (Path2)**	0.58	0.05	0.45	0.65	0.63	0.06	0.50	0.74
**ASV (Path3)**	0.10	0.06	0.01	0.24	0.17	0.08	0.04	0.33
**CORT (Path4)**	0.41	0.09	0.22	0.56	0.54	0.10	0.30	0.70

4. Bayesian Structural Equation Modeling (SEM) diagnostics of 16S rRNA diversity measurements - model results for Haptoglobin immune assay

Table S13. Shannon diversity index

**Group-Level Effects:**							
**nest (Number of levels: 23)**							
	**Estimate**	**Est.Error**	**l-95% CI**	**u-95% CI**	**Rhat**	**Bulk_ESS**	**Tail_ESS**
sd(BCI_Intercept)	0.12	0.04	0.05	0.20	1.00	46577	40288
sd(Haptoglobin_Intercept)	0.06	0.03	0.00	0.12	1.00	37280	61394
sd(Shannon_Intercept)	0.05	0.04	0.00	0.13	1.00	70193	93652
sd(CORT_Intercept)	0.04	0.02	0.00	0.09	1.00	49165	80164
**nest:ring_number (Number of levels: 43)**							
	**Estimate**	**Est.Error**	**l-95% CI**	**u-95% CI**	**Rhat**	**Bulk_ESS**	**Tail_ESS**
sd(BCI_Intercept)	0.05	0.04	0.00	0.13	1.00	34914	59787
sd(Haptoglobin_Intercept)	0.04	0.02	0.00	0.09	1.00	52859	91654
sd(Shannon_Intercept)	0.04	0.03	0.00	0.11	1.00	90472	99033
sd(CORT_Intercept)	0.03	0.02	0.00	0.08	1.00	49587	92268
**Population-Level Effects:**							
	**Estimate**	**Est.Error**	**l-95% CI**	**u-95% CI**	**Rhat**	**Bulk_ESS**	**Tail_ESS**
BCI_Intercept	0.65	0.12	0.42	0.88	1.00	166370	152064
Haptoglobin_Intercept	0.07	0.09	-0.12	0.25	1.00	207964	151016
shannon_Intercept	0.58	0.14	0.31	0.84	1.00	214929	148041
CORT_Intercept	0.59	0.04	0.51	0.69	1.00	170215	163200
BCI_Shannon	0.18	0.09	0.01	0.35	1.00	279225	157629
BCI_CORT	-0.43	0.15	-0.71	-0.14	1.00	176535	155057
BCI_Haptoglobin	0.20	0.16	-0.12	0.51	1.00	146181	141461
BCI_Age	-0.11	0.12	-0.34	0.12	1.00	150130	154761
Haptoglobin_Shannon	0.07	0.07	-0.07	0.21	1.00	306014	156540
Haptoglobin_CORT	0.02	0.12	-0.20	0.25	1.00	196783	158952
Haptoglobin_Age	0.03	0.09	-0.15	0.21	1.00	210761	157411
Shannon_CORT	0.02	0.19	-0.35	0.38	1.00	212876	160860
Shannon_Age	-0.07	0.15	-0.37	0.23	1.00	220302	164092
CORT_Age	-0.47	0.08	-0.63	-0.32	1.00	151514	161846
**Family Specific Parameters:**							
	**Estimate**	**Est.Error**	**l-95% CI**	**u-95% CI**	**Rhat**	**Bulk_ESS**	**Tail_ESS**
sigma_BCI	0.15	0.02	0.12	0.18	1.00	83069	120711
sigma_Haptoglobin	0.13	0.01	0.11	0.16	1.00	107411	142534
sigma_Shannon	0.22	0.02	0.19	0.27	1.00	237812	149810

sigma_CORT	0.14	0.01	0.11	0.17	1.00	107827	137420
alpha_CORT	4.49	2.73	-0.60	10.26	1.00	120661	143627

Table S14. Faith PD

**Group-Level Effects:**							
**nest (Number of levels: 23)**							
	**Estimate**	**Est.Error**	**l-95% CI**	**u-95% CI**	**Rhat**	**Bulk_ESS**	**Tail_ESS**
sd(BCI_Intercept)	0.12	0.04	0.04	0.20	1.00	38682	31898
sd(Haptoglobin_Intercept)	0.05	0.03	0.00	0.12	1.00	38548	61800
sd(FaithPD_Intercept)	0.05	0.03	0.00	0.12	1.00	60992	88316
sd(CORT_Intercept)	0.04	0.02	0.00	0.09	1.00	47206	77017
**nest:ring_number (Number of levels: 43)**							
	**Estimate**	**Est.Error**	**l-95% CI**	**u-95% CI**	**Rhat**	**Bulk_ESS**	**Tail_ESS**
sd(BCI_Intercept)	0.06	0.04	0.00	0.14	1.00	29673	54217
sd(Haptoglobin_Intercept)	0.03	0.02	0.00	0.09	1.00	56438	92926
sd(FaithPD_Intercept)	0.04	0.03	0.00	0.11	1.00	63496	88641
sd(CORT_Intercept)	0.03	0.02	0.00	0.08	1.00	46184	90112
**Population-Level Effects:**							
	**Estimate**	**Est.Error**	**l-95% CI**	**u-95% CI**	**Rhat**	**Bulk_ESS**	**Tail_ESS**
BCI_Intercept	0.70	0.12	0.46	0.94	1.00	160066	150464
Haptoglobin_Intercept	0.12	0.09	-0.07	0.30	1.00	183369	150088
FaithPD_Intercept	0.48	0.12	0.25	0.70	1.00	183336	149744
CORT_Intercept	0.59	0.04	0.51	0.69	1.00	160318	156040
BCI_FaithPD	0.11	0.11	-0.10	0.32	1.00	195841	156807
BCI_CORT	-0.43	0.15	-0.72	-0.14	1.00	166981	154805
BCI_Haptoglobin	0.23	0.17	-0.10	0.55	1.00	127842	133346
BCI_Age	-0.11	0.12	-0.34	0.14	1.00	136464	149465
Haptoglobin_FaithPD	-0.02	0.09	-0.19	0.15	1.00	230578	153645
Haptoglobin_CORT	0.03	0.12	-0.20	0.26	1.00	185160	157469
Haptoglobin_Age	0.02	0.09	-0.16	0.21	1.00	193232	157016
FaithPD_CORT	0.07	0.16	-0.25	0.38	1.00	183577	158376
FaithPD_Age	-0.16	0.13	-0.42	0.09	1.00	195686	159848
CORT_Age	-0.47	0.08	-0.63	-0.32	1.00	143907	154942

**Family Specific Parameters:**							
	**Estimate**	**Est.Error**	**l-95% CI**	**u-95% CI**	**Rhat**	**Bulk_ESS**	**Tail_ESS**
sigma_BCI	0.15	0.02	0.12	0.19	1.00	69276	114960
sigma_Haptoglobin	0.13	0.01	0.11	0.16	1.00	112819	138462
sigma_FaithPD	0.19	0.02	0.16	0.22	1.00	172827	151782
sigma_CORT	0.14	0.01	0.11	0.17	1.00	99573	138620
alpha_CORT	4.51	2.71	-0.57	10.29	1.00	110645	132494

Table S15. N° of Observed ASV's

**Group-Level Effects:**							
**nest (Number of levels: 23)**							
	**Estimate**	**Est.Error**	**l-95% CI**	**u-95% CI**	**Rhat**	**Bulk_ESS**	**Tail_ESS**
sd(BCI_Intercept)	0.12	0.04	0.04	0.20	1.00	49217	43984
sd(Haptoglobin_Intercept)	0.06	0.03	0.00	0.12	1.00	43823	78048
sd(ASV_Intercept)	0.04	0.03	0.00	0.10	1.00	73534	108245
sd(CORT_Intercept)	0.04	0.02	0.00	0.09	1.00	55507	95386
**nest:ring_number (Number of levels: 43)**							
	**Estimate**	**Est.Error**	**l-95% CI**	**u-95% CI**	**Rhat**	**Bulk_ESS**	**Tail_ESS**
sd(BCI_Intercept)	0.06	0.04	0.00	0.14	1.00	36515	64074
sd(Haptoglobin_Intercept)	0.03	0.02	0.00	0.09	1.00	64382	105246
sd(ASV_Intercept)	0.03	0.02	0.00	0.08	1.00	98168	110066
sd(CORT_Intercept)	0.03	0.02	0.00	0.08	1.00	58612	101913
**Population-Level Effects:**							
	**Estimate**	**Est.Error**	**l-95% CI**	**u-95% CI**	**Rhat**	**Bulk_ESS**	**Tail_ESS**
BCI_Intercept	0.69	0.12	0.45	0.92	1.00	249579	154242
Haptoglobin_Intercept	0.10	0.09	-0.09	0.28	1.00	296794	155887
ASV_Intercept	0.40	0.10	0.20	0.60	1.00	318155	154810
CORT_Intercept	0.59	0.04	0.51	0.69	1.00	224297	172718
BCI_ASV	0.17	0.12	-0.06	0.40	1.00	320964	155417
BCI_CORT	-0.43	0.15	-0.72	-0.14	1.00	257536	161108
BCI_Haptoglobin	0.22	0.16	-0.11	0.54	1.00	170007	151221
BCI_Age	-0.09	0.12	-0.33	0.15	1.00	202081	155126
Haptoglobin_ASV	0.02	0.10	-0.17	0.22	1.00	336553	157222

Group-Level Effects:							
Haptoglobin_CORT	0.02	0.12	-0.21	0.25	1.00	285478	164665
Haptoglobin_Age	0.03	0.10	-0.16	0.22	1.00	297820	167261
ASV_CORT	0.06	0.14	-0.22	0.33	1.00	289168	164095
ASV_Age	-0.17	0.11	-0.40	0.05	1.00	304712	167802
CORT_Age	-0.47	0.08	-0.63	-0.32	1.00	198304	169954
**Family Specific Parameters:**							
	**Estimate**	**Est.Error**	**l-95% CI**	**u-95% CI**	**Rhat**	**Bulk_ESS**	**Tail_ESS**
sigma_BCI	0.15	0.02	0.12	0.19	1.00	80124	128574
sigma_Haptoglobin	0.13	0.01	0.11	0.16	1.00	130042	145658
sigma_ASV	0.17	0.01	0.14	0.20	1.00	279694	157778
sigma_CORT	0.14	0.01	0.11	0.17	1.00	129980	148975
alpha_CORT	4.51	2.73	-0.59	10.29	1.00	146675	154517

Table S16. Bayes R2 for each diversity measurement

	R2m	Est.Error	Q2.5	Q97.5	R2c	Est.Error	Q2.5	Q97.5
**BCI (Path1)**	0.18	0.06	0.07	0.29	0.52	0.08	0.33	0.66
**Haptoglobin (Path2)**	0.05	0.03	0.00	0.13	0.24	0.10	0.06	0.43
**Shannon (Path3)**	0.03	0.03	0.00	0.11	0.12	0.07	0.02	0.28
**CORT (Path4)**	0.36	0.08	0.20	0.52	0.47	0.11	0.25	0.66
	**R2m**	**Est.Error**	**Q2.5**	**Q97.5**	**R2c**	**Est.Error**	**Q2.5**	**Q97.5**
**BCI (Path1)**	0.16	0.06	0.05	0.27	0.50	0.09	0.30	0.65
**Haptoglobin (Path2)**	0.04	0.03	0.00	0.11	0.22	0.10	0.05	0.41
**Faith PD (Path3)**	0.08	0.05	0.01	0.20	0.19	0.09	0.05	0.37
**CORT (Path4)**	0.36	0.08	0.20	0.52	0.47	0.11	0.25	0.66
	**R2m**	**Est.Error**	**Q2.5**	**Q97.5**	**R2c**	**Est.Error**	**Q2.5**	**Q97.5**
**BCI (Path1)**	0.16	0.06	0.06	0.28	0.50	0.09	0.31	0.65
**Haptoglobin (Path2)**	0.04	0.03	0.00	0.11	0.23	0.10	0.05	0.42
**ASV (Path3)**	0.10	0.06	0.01	0.22	0.18	0.08	0.05	0.34
**CORT (Path4)**	0.36	0.08	0.20	0.52	0.47	0.11	0.25	0.66

5. Bayesian Structural Equation Modeling (SEM) diagnostics of 28S rRNA diversity measurements - model results for Haptoglobin immune assay

Table S17. Shannon diversity index

**Group-Level Effects:**							
**nest (Number of levels: 23)**							
	**Estimate**	**Est.Error**	**l-95% CI**	**u-95% CI**	**Rhat**	**Bulk_ESS**	**Tail_ESS**
sd(BCI_Intercept)	0.13	0.04	0.05	0.22	1.00	42951	37948
sd(Haptoglobin_Intercept)	0.05	0.03	0.00	0.11	1.00	50319	76803
sd(Shannon_Intercept)	0.05	0.04	0.00	0.14	1.00	85975	99903
sd(CORT_Intercept)	0.05	0.03	0.00	0.12	1.00	43080	63799
**nest:ring_number (Number of levels: 41)**							
	**Estimate**	**Est.Error**	**l-95% CI**	**u-95% CI**	**Rhat**	**Bulk_ESS**	**Tail_ESS**
sd(BCI_Intercept)	0.05	0.04	0.00	0.13	1.00	42050	65893
sd(Haptoglobin_Intercept)	0.04	0.03	0.00	0.09	1.00	61510	96100
sd(Shannon_Intercept)	0.05	0.04	0.00	0.13	1.00	80886	90962
sd(CORT_Intercept)	0.04	0.03	0.00	0.09	1.00	49853	91266
**Population-Level Effects:**							
	**Estimate**	**Est.Error**	**l-95% CI**	**u-95% CI**	**Rhat**	**Bulk_ESS**	**Tail_ESS**
BCI_Intercept	0.79	0.14	0.52	1.05	1.00	177015	149832
Haptoglobin_Intercept	0.04	0.11	-0.17	0.26	1.00	178238	149776
shannon_Intercept	0.77	0.15	0.47	1.07	1.00	193181	148698
CORT_Intercept	0.62	0.05	0.53	0.72	1.00	202412	169172
BCI_Shannon	-0.09	0.09	-0.27	0.09	1.00	256177	154358
BCI_CORT	-0.40	0.16	-0.71	-0.09	1.00	166515	152065
BCI_Haptoglobin	0.34	0.17	-0.01	0.67	1.00	131149	122600
BCI_Age	-0.15	0.13	-0.40	0.11	1.00	154134	145350
Haptoglobin_Shannon	0.08	0.08	-0.07	0.23	1.00	266918	156618
Haptoglobin_CORT	0.03	0.13	-0.22	0.28	1.00	185576	160109
Haptoglobin_Age	0.05	0.10	-0.16	0.25	1.00	193838	161802
Shannon_CORT	-0.07	0.21	-0.48	0.34	1.00	196076	154250
Shannon_Age	-0.26	0.17	-0.60	0.07	1.00	202369	161208
CORT_Age	-0.50	0.08	-0.67	-0.34	1.00	178286	162816
**Family Specific Parameters:**							

	**Estimate**	**Est.Error**	**l-95% CI**	**u-95% CI**	**Rhat**	**Bulk_ESS**	**Tail_ESS**
sigma_BCI	0.15	0.02	0.12	0.19	1.00	80726	110024
sigma_Haptoglobin	0.14	0.01	0.11	0.17	1.00	130548	148475
sigma_Shannon	0.24	0.02	0.19	0.28	1.00	221222	146208
sigma_CORT	0.14	0.02	0.11	0.17	1.00	107657	138376
alpha_CORT	3.12	2.72	-1.74	8.98	1.00	95525	115777

Table S18. Faith PD

**Group-Level Effects:**							
**nest (Number of levels: 23)**							
	**Estimate**	**Est.Error**	**l-95% CI**	**u-95% CI**	**Rhat**	**Bulk_ESS**	**Tail_ESS**
sd(BCI_Intercept)	0.13	0.04	0.05	0.22	1.00	45351	40452
sd(Haptoglobin_Intercept)	0.05	0.03	0.00	0.11	1.00	51291	79207
sd(FaithPD_Intercept)	0.03	0.03	0.00	0.10	1.00	101230	101146
sd(CORT_Intercept)	0.05	0.03	0.00	0.12	1.00	41187	66489
**nest:ring_number (Number of levels: 41)**							
	**Estimate**	**Est.Error**	**l-95% CI**	**u-95% CI**	**Rhat**	**Bulk_ESS**	**Tail_ESS**
sd(BCI_Intercept)	0.05	0.03	0.00	0.13	1.00	42197	63897
sd(Haptoglobin_Intercept)	0.04	0.03	0.00	0.10	1.00	60837	92942
sd(FaithPD_Intercept)	0.04	0.03	0.00	0.10	1.00	95660	94232
sd(CORT_Intercept)	0.04	0.03	0.00	0.09	1.00	50249	93337
**Population-Level Effects:**							
	**Estimate**	**Est.Error**	**l-95% CI**	**u-95% CI**	**Rhat**	**Bulk_ESS**	**Tail_ESS**
BCI_Intercept	0.79	0.13	0.54	1.04	1.00	164304	146946
Haptoglobin_Intercept	0.09	0.10	-0.12	0.29	1.00	180969	143777
FaithPD_Intercept	0.45	0.12	0.20	0.69	1.00	208782	149273
CORT_Intercept	0.62	0.05	0.53	0.72	1.00	208633	166641
BCI_FaithPD	-0.16	0.11	-0.38	0.05	1.00	257166	152653
BCI_CORT	-0.41	0.16	-0.72	-0.10	1.00	171235	151359
BCI_Haptoglobin	0.33	0.17	-0.02	0.66	1.00	137582	124258
BCI_Age	-0.17	0.13	-0.42	0.09	1.00	155648	144723
Haptoglobin_FaithPD	0.04	0.10	-0.15	0.23	1.00	269140	154322
Haptoglobin_CORT	0.02	0.13	-0.23	0.28	1.00	189700	156382

Haptoglobin_Age	0.04	0.11	-0.17	0.25	1.00	198629	154843
FaithPD_CORT	-0.07	0.17	-0.40	0.26	1.00	210016	159100
FaithPD_Age	-0.28	0.14	-0.55	-0.01	1.00	214852	156644
CORT_Age	-0.50	0.08	-0.67	-0.34	1.00	177992	158833
**Family Specific Parameters:**							
	**Estimate**	**Est.Error**	**l-95% CI**	**u-95% CI**	**Rhat**	**Bulk_ESS**	**Tail_ESS**
sigma_BCI	0.15	0.02	0.12	0.19	1.00	81762	109599
sigma_Haptoglobin	0.14	0.01	0.11	0.17	1.00	129246	148539
sigma_FaithPD	0.19	0.02	0.16	0.23	1.00	266445	144974
sigma_CORT	0.14	0.02	0.11	0.17	1.00	109605	136275
alpha_CORT	3.13	2.71	-1.74	8.96	1.00	97554	117030

Table S19. N° of observed ASV's

**Group-Level Effects:**							
**~nest (Number of levels: 23)**							
	**Estimate**	**Est.Error**	**l-95% CI**	**u-95% CI**	**Rhat**	**Bulk_ESS**	**Tail_ESS**
sd(BCI_Intercept)	0.13	0.04	0.05	0.22	1.00	48956	43678
sd(Haptoglobin_Intercept)	0.05	0.03	0.00	0.11	1.00	51356	83029
sd(ASV_Intercept)	0.03	0.02	0.00	0.09	1.00	121055	106693
sd(CORT_Intercept)	0.05	0.03	0.00	0.12	1.00	46631	76304
**nest:ring_number (Number of levels: 41)**							
	**Estimate**	**Est.Error**	**l-95% CI**	**u-95% CI**	**Rhat**	**Bulk_ESS**	**Tail_ESS**
sd(BCI_Intercept)	0.05	0.03	0.00	0.13	1.00	45517	68906
sd(Haptoglobin_Intercept)	0.04	0.03	0.00	0.10	1.00	62403	101415
sd(ASV_Intercept)	0.04	0.03	0.00	0.11	1.00	92477	104228
sd(CORT_Intercept)	0.04	0.03	0.00	0.09	1.00	52226	93595
**Population-Level Effects:**							
	**Estimate**	**Est.Error**	**l-95% CI**	**u-95% CI**	**Rhat**	**Bulk_ESS**	**Tail_ESS**
BCI_Intercept	0.80	0.13	0.54	1.05	1.00	207560	156305
Haptoglobin_Intercept	0.08	0.11	-0.13	0.29	1.00	225466	154892
ASV_Intercept	0.52	0.12	0.27	0.77	1.00	259536	150683
CORT_Intercept	0.62	0.05	0.53	0.72	1.00	247458	171992

BCI_ASV	-0.15	0.11	-0.36	0.06	1.00	326297	160195
BCI_CORT	-0.42	0.16	-0.72	-0.10	1.00	206788	157745
BCI_Haptoglobin	0.34	0.17	-0.01	0.67	1.00	149067	124855
BCI_Age	-0.17	0.13	-0.42	0.10	1.00	182753	148280
Haptoglobin_ASV	0.05	0.09	-0.13	0.24	1.00	339562	157186
Haptoglobin_CORT	0.03	0.13	-0.23	0.28	1.00	227703	164385
Haptoglobin_Age	0.04	0.11	-0.17	0.25	1.00	236824	164963
ASV_CORT	-0.11	0.17	-0.44	0.23	1.00	250166	163417
ASV_Age	-0.28	0.14	-0.56	-0.01	1.00	253690	160598
CORT_Age	-0.50	0.08	-0.67	-0.34	1.00	211111	163889
**Family Specific Parameters:**							
	**Estimate**	**Est.Error**	**l-95% CI**	**u-95% CI**	**Rhat**	**Bulk_ESS**	**Tail_ESS**
sigma_BCI	0.15	0.02	0.12	0.19	1.00	85554	115319
sigma_Haptoglobin	0.14	0.01	0.11	0.17	1.00	135435	151746
sigma_ASV	0.19	0.02	0.16	0.23	1.00	292603	149156
sigma_CORT	0.14	0.02	0.11	0.17	1.00	120035	144255
alpha_CORT	3.12	2.72	-1.74	8.94	1.00	108540	126059

Table S20. Bayes R2 for each diversity measurement

	R2m	Est.Error	Q2.5	Q97.5	R2c	Est.Error	Q2.5	Q97.5
**BCI (Path1)**	0.17	0.06	0.06	0.28	0.51	0.09	0.29	0.66
**Haptoglobin (Path2)**	0.06	0.04	0.01	0.16	0.23	0.10	0.05	0.43
**Shannon (Path3)**	0.08	0.05	0.00	0.20	0.16	0.08	0.03	0.33
**CORT (Path4)**	0.41	0.09	0.22	0.56	0.54	0.10	0.30	0.70
	**R2m**	**Est.Error**	**Q2.5**	**Q97.5**	**R2c**	**Est.Error**	**Q2.5**	**Q97.5**
**BCI (Path1)**	0.18	0.06	0.06	0.29	0.52	0.09	0.30	0.66
**Haptoglobin (Path2)**	0.05	0.03	0.00	0.13	0.22	0.10	0.05	0.43
**Faith PD (Path3)**	0.11	0.06	0.01	0.25	0.17	0.07	0.04	0.33
**CORT (Path4)**	0.41	0.09	0.22	0.56	0.54	0.10	0.30	0.70
	**R2m**	**Est.Error**	**Q2.5**	**Q97.5**	**R2c**	**Est.Error**	**Q2.5**	**Q97.5**
**BCI (Path1)**	0.17	0.06	0.06	0.29	0.52	0.09	0.30	0.67
**Haptoglobin (Path2)**	0.05	0.04	0.00	0.14	0.22	0.10	0.05	0.43
**ASV (Path3)**	0.10	0.06	0.01	0.24	0.16	0.08	0.04	0.33

	R2m	Est.Error	Q2.5	Q97.5	R2c	Est.Error	Q2.5	Q97.5
**CORT (Path4)**	0.41	0.09	0.22	0.56	0.54	0.10	0.30	0.70

Figure S5. Differential abundance analysis results for each of the variables in study - ANCOM-BC2 model incorporating the latent variable *Immunity*

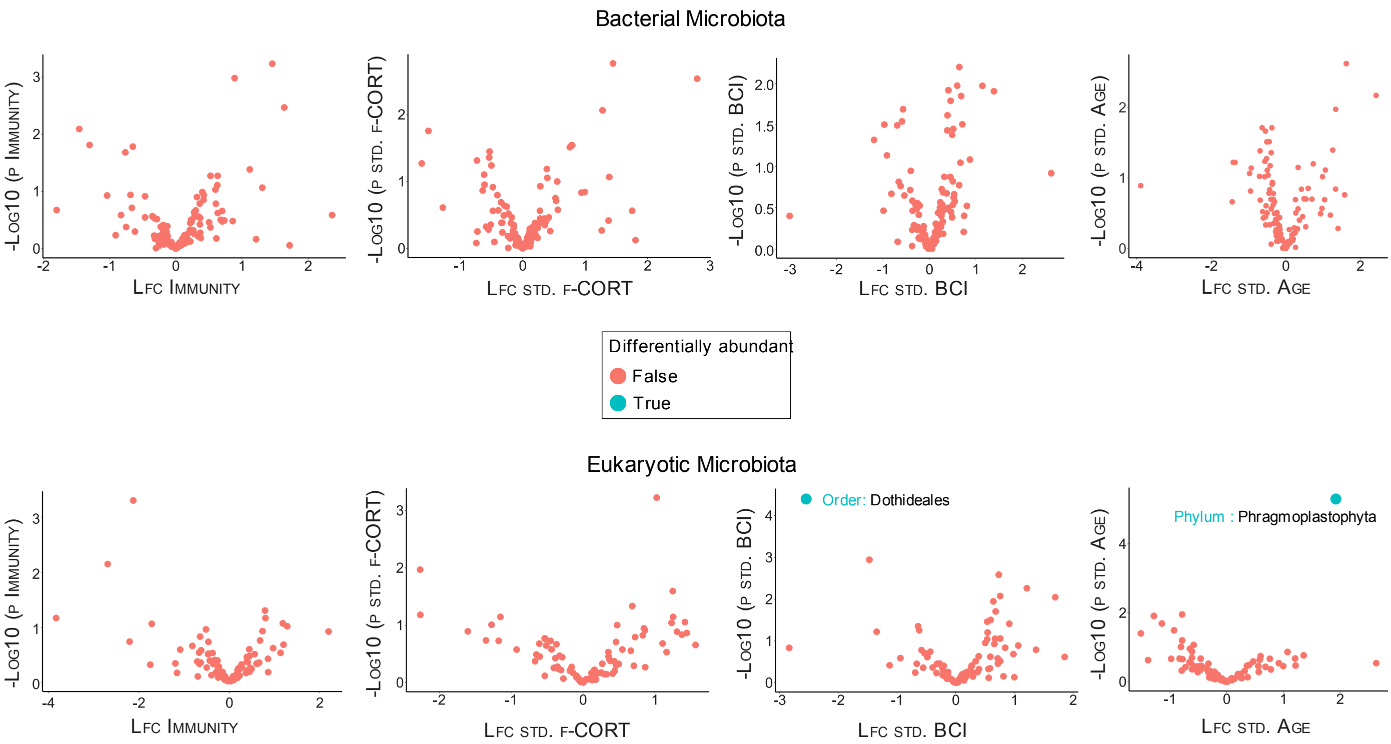

Table S21. Sensitivity analysis for the two deferentially abundant taxa

**Taxon**	**lfc (std.BCI)**	**se (std.BCI)**	**W (std.BCI)**	**p (std.BCI)**	**q (std.BCI)**	**diff (std.BCI)**	**passed_ss (std.BCI)**
d__Eukaryota;p_Ascomycota; c_Dothideomycetes;o_Dothideales; f_Dothideales;g_Dothideales; s_Hormonema_carpetanum	-2.60	0.18	-14.12	0.00	0.01	TRUE	TRUE
**Taxon**	**lfc (std.Age)**	**se (std.Age)**	**W (std.Age)**	**p (std.Age)**	**q (std.Age)**	**diff (std.Age)**	**passed_ss (std.Age)**
d__Eukaryota;p_Phragmoplastophyta; c_Phragmoplastophyta;o_Phragmoplastophyta; f_Phragmoplastophyta; g_Phragmoplastophyta;s_Pinus_taeda	1.90	0.23	8.27	0.00	0.00	TRUE	FALSE

Figure S6. Differential abundance analysis results for each of the variables in study - ANCOM-BC2 model incorporating Haptoglobin immune assay

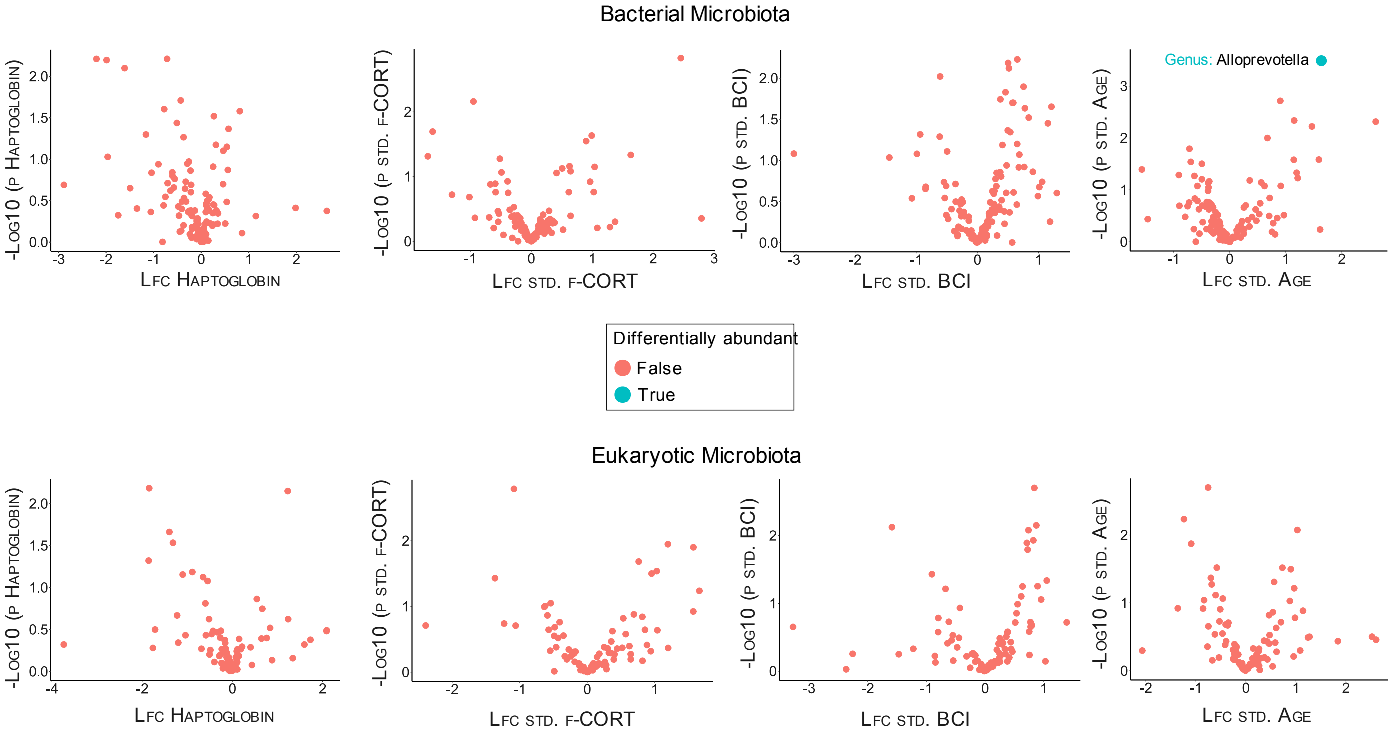

Table S22 Sensitivity analysis for the deferentially abundant taxa

Taxon	lfc (std.Age)	se (std.Age)	W (std.Age)	p (std.Age)	q (std.Age)	diff (std.Age)	passed_ss (std.Age)
d__Bacteria;p_Bacteroidota; c_Bacteroidia;o_Bacteroidales; f_Prevotellaceae;g_Alloprevotella;s_Alloprevotella_rava	1.60	0.18	8.92	0.00	0.05	TRUE	FALSE

Figure S7. Bayesian structural equation models for the different bacterial diversity measures with results from each immune assay superimposed onto each diagram

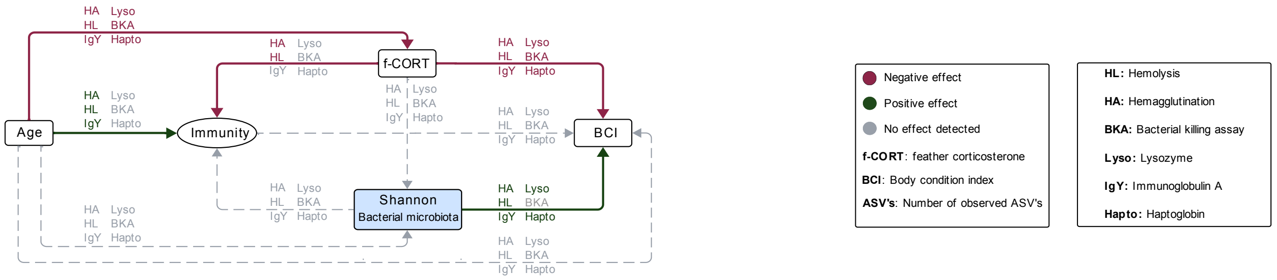

Figure S8. Bayesian structural equation models for the different eukaryotic diversity measures with results from each immune assay superimposed onto each diagram

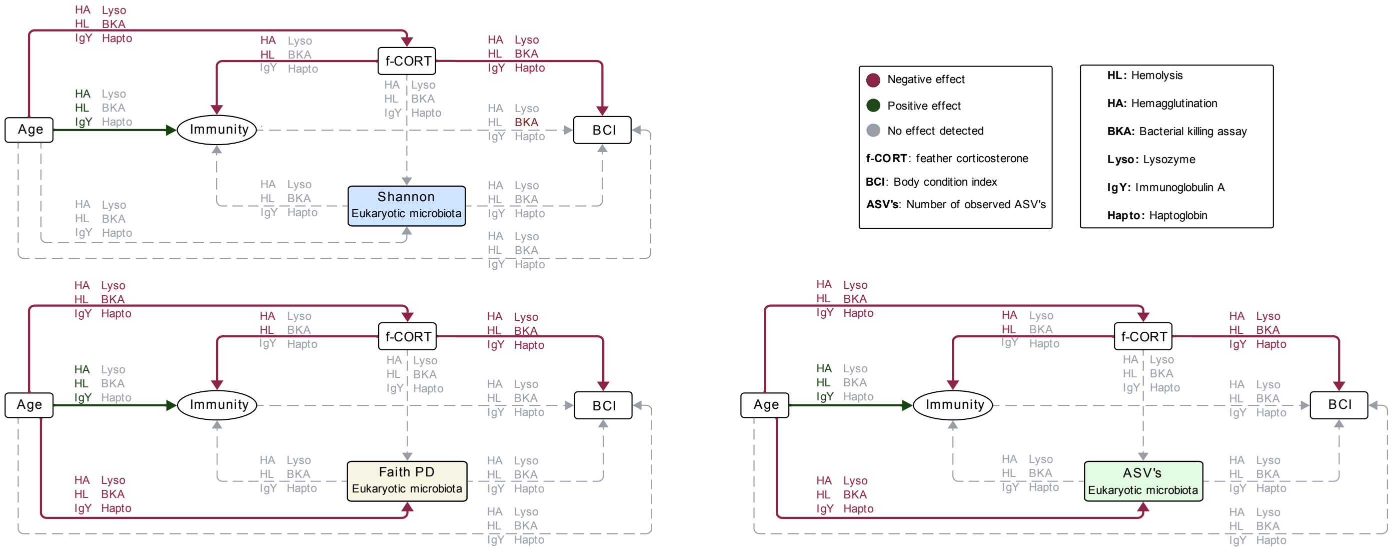

Supplement: Supplementary file 1 [file Data_Sheet_1.pdf]
