# Supplementary material for: The gut microbiota-immune-brain axis in a wild vertebrate: dynamic interactions and health impacts
Source: Front Microbiol. 2024 Sep 10;15:1413976. doi: 10.3389/fmicb.2024.1413976 (PMC11420037; doi:10.3389/fmicb.2024.1413976)
Supplement: Supplementary file 5 [file Data_Sheet_5.PDF]

Differential Abundant Analysis with ANCOM-BC2

Pipeline adapted from https://bioconductor.org/packages/release/bioc/vignettes/ANCOMBC/inst/doc/ANCOMBC2.html

Table of Contents

- Differential Abundant Analysis with ANCOM-BC2
  - Table of Contents
- A) DAA 16s rRNA
  - 1. Read in the data
  - 2. Run ANCOM-BC2
    - 2.1 ANCOM-BC2 at ASV level
      - 2.1.1 ANCOM-BC2 primary analysis
      - 2.1.2 Sensitivity scores
      - 2.2.3 Plot ANCOM-BC2 results
- B) DAA 28s rRNA
  - 1. Read in the data
  - 2. Run ANCOM-BC2
    - 2.1 ANCOM-BC2 at ASV level
      - 2.1.1 ANCOM-BC2 primary analysis
      - 2.1.2 Sensitivity scores
      - 2.2.3 Plot ANCOM-BC2 results

A) DAA 16s rRNA

1. Read in the data

```
# Load libraries
library(phyloseq)
library(tidyverse)
library(janitor)
library(microbiome)
library(ANCOMBC)
library(ggrepel)

# read in the phyloseq object

ps_immune <- readRDS("ps_immune.rds")
```

2. Run ANCOM-BC2

2.1 ANCOM-BC2 at ASV level

```
# Diferential abundace analysis on final model.

set.seed(123)
output_immunity = ancombc2(data = ps_immune, assay_name = "counts", tax_level = NULL,
                        fix_formula = "std_bci_two + std_cort + pred_immunity + std_age",
rand_formula = "(1|nest/ring_number)",
                        p_adj_method = "holm", pseudo = 0, pseudo_sens = TRUE,
                        prv_cut = 0.10, lib_cut = 0, s0_perc = 0.05,
                        #group = NULL, struc_zero = TRUE, neg_lb = TRUE,
                        alpha = 0.05, n_cl = 2, verbose = TRUE,
                        #global = TRUE, pairwise = TRUE, dunnet = TRUE, trend = TRUE,
                        iter_control = list(tol = 1e-2, max_iter = 50, verbose = TRUE),
                        em_control = list(tol = 1e-5, max_iter = 100),
                        lme_control = lme4::lmerControl(optimizer ="Nelder_Mead"),
                        mdfdr_control = list(fwer_ctrl_method = "holm", B = 100),
                        trend_control = list(contrast = list(matrix(c(1, 0, -1, 1),
                        nrow = 2, byrow = TRUE), matrix(c(-1, 0, 1, -1), nrow = 2,
                        byrow = TRUE)), node = list(2, 2), solver = "ECOS", B = 1000))

#run the same model for haptoglobin
output_hapto = ancombc2(data = ps_immune, assay_name = "counts", tax_level = NULL,
                        fix_formula = "std_bci_two + std_cort + std_hapto + std_age",
rand_formula = "(1|nest/ring_number)",
                        p_adj_method = "holm", pseudo = 0, pseudo_sens = TRUE,
                        prv_cut = 0.10, lib_cut = 0, s0_perc = 0.05,
                        #group = NULL, struc_zero = TRUE, neg_lb = TRUE,
                        alpha = 0.05, n_cl = 2, verbose = TRUE,
                        #global = TRUE, pairwise = TRUE, dunnet = TRUE, trend = TRUE,
                        iter_control = list(tol = 1e-2, max_iter = 50, verbose = TRUE),
                        em_control = list(tol = 1e-5, max_iter = 100),
                        lme_control = lme4::lmerControl(optimizer ="Nelder_Mead"),
                        mdfdr_control = list(fwer_ctrl_method = "holm", B = 100),
                        trend_control = list(contrast = list(matrix(c(1, 0, -1, 1),
                        nrow = 2, byrow = TRUE), matrix(c(-1, 0, 1, -1), nrow = 2,
                        byrow = TRUE)), node = list(2, 2), solver = "ECOS", B = 1000))
```

2.1.1 ANCOM-BC2 primary analysis

```
res_prim_immunity = output_immunity$res

res_prim_hapto = output_hapto$res

 # Check taxonomy of DAA taxa
 DAA_hapto <- c("86f88dec7777f0258548b6bdb3ea53c9")
 DAA_hapto_ps <- prune_taxa(taxa_names(ps_immune) %in% DAA_hapto, ps_immune)
 DAA_hapto_ps@tax_table
                                   #Kingdom      Phylum       Class         Order
Family           Genus
 #86f88dec7777f0258548b6bdb3ea53c9 "Bacteria" "Bacteroidota" "Bacteroidia" "Bacteroidales"
"Prevotellaceae" "Alloprevotella"
#Species
#"Alloprevotella_rava"
```

No deferentially abundant ASVs found for models with latente variable Immunity

One deferentially abundant ASVs (with age) found for models with haptoglobin did not pass the sensitivity analysis

2.1.2 Sensitivity scores

ANCOM-BC2 uses a sensitivity analysis to assess the impact of different pseudo-counts on zero counts for each taxon. The sensitivity score is determined by performing linear regression models on the bias-corrected log abundance table using various pseudo-counts and calculating the proportion of times the p-value exceeds the significance level (alpha). This helps identify taxa that are not sensitive to the pseudo-count addition, ensuring robustness in the analysis.

```
tab_sens_immunity = output_immunity$ss_tab

tab_sens_hapto = output_hapto$ss_tab
```

2.2.3 Plot ANCOM-BC2 results

```
# Volcano plots - immunity
volc_immunity_bci <- ggplot(data=res_prim_immunity, aes(x=lfc_std_bci_two, y=-
log10(p_std_bci_two), col=diff_std_bci_two)) + geom_point(size=3) +
   geom_text_repel(aes(label = ifelse(diff_std_bci_two, taxon, ""))) +
   theme_classic()+
  labs(x = "Lfc std. BCI", y = "-Log10 (p std. BCI)") +
  theme(axis.text.x = element_text(size = 16),  # Adjust the size as needed
        axis.text.y = element_text(size = 16))+
  theme(axis.title.x = element_text(size = 16),  # Adjust the size as needed
        axis.title.y = element_text(size = 16))+
  theme(axis.title.x = element_text(margin = margin(t = 13)))+
  theme(axis.title.y = element_text(margin = margin(r = 14)))+
  theme(legend.position = "none")+
  theme(text = element_text(family = "Arial"))
 volc_immunity_bci

 volc_immunity_immunity <- ggplot(data=res_prim_immunity, aes(x=lfc_pred_immunity, y=-
log10(p_pred_immunity), col=diff_pred_immunity)) + geom_point(size=3) +
   geom_text_repel(aes(label = ifelse(diff_pred_immunity, taxon, ""))) +
   theme_classic()+
   labs(x = "Lfc Immunity", y = "-Log10 (p Immunity)") +
   theme(axis.text.x = element_text(size = 16),  # Adjust the size as needed
         axis.text.y = element_text(size = 16))+
   theme(axis.title.x = element_text(size = 16),  # Adjust the size as needed
         axis.title.y = element_text(size = 16))+
   theme(axis.title.x = element_text(margin = margin(t = 13)))+
   theme(axis.title.y = element_text(margin = margin(r = 14)))+
   theme(legend.position = "none")+
   theme(text = element_text(family = "Arial"))
 volc_immunity_immunity

 volc_immunity_cort <- ggplot(data=res_prim_immunity, aes(x=lfc_std_cort, y=-log10(p_std_cort),
col=diff_std_cort)) + geom_point(size=3) +
   geom_text_repel(aes(label = ifelse(diff_std_cort, taxon, ""))) +
   theme_classic()+
   labs(x = "Lfc std. CORT", y = "-Log10 (p std. CORT)") +
   theme(axis.text.x = element_text(size = 16),  # Adjust the size as needed
         axis.text.y = element_text(size = 16))+
   theme(axis.title.x = element_text(size = 16),  # Adjust the size as needed
```

```
         axis.title.y = element_text(size = 16))+
   theme(axis.title.x = element_text(margin = margin(t = 13)))+
   theme(axis.title.y = element_text(margin = margin(r = 14)))+
   theme(legend.position = "none")+
   theme(text = element_text(family = "Arial"))
 volc_immunity_cort

 volc_immunity_age <- ggplot(data=res_prim_immunity, aes(x=lfc_std_age, y=-log10(p_std_age),
col=diff_std_age)) + geom_point(size=3) +
   geom_text_repel(aes(label = ifelse(diff_std_age, taxon, ""))) +
   theme_classic()+
   labs(x = "Lfc std. Age", y = "-Log10 (p std. Age)") +
   theme(axis.text.x = element_text(size = 16),  # Adjust the size as needed
         axis.text.y = element_text(size = 16))+
   theme(axis.title.x = element_text(size = 16),  # Adjust the size as needed
         axis.title.y = element_text(size = 16))+
   theme(axis.title.x = element_text(margin = margin(t = 13)))+
   theme(axis.title.y = element_text(margin = margin(r = 14)))+
   theme(text = element_text(family = "Arial"))
 volc_immunity_age


# Volcano plots - haptoglobin

volc_hapto_bci <- ggplot(data=res_prim_hapto, aes(x=lfc_std_bci_two, y=-log10(p_std_bci_two),
col=diff_std_bci_two)) + geom_point(size=3) +
   geom_text_repel(aes(label = ifelse(diff_std_bci_two, taxon, ""))) +
   theme_classic()+
  labs(x = "Lfc std. BCI", y = "-Log10 (p std. BCI)") +
  theme(axis.text.x = element_text(size = 16),  # Adjust the size as needed
        axis.text.y = element_text(size = 16))+
  theme(axis.title.x = element_text(size = 16),  # Adjust the size as needed
        axis.title.y = element_text(size = 16))+
  theme(axis.title.x = element_text(margin = margin(t = 13)))+
  theme(axis.title.y = element_text(margin = margin(r = 14)))+
  theme(legend.position = "none")+
  theme(text = element_text(family = "Arial"))
 volc_hapto_bci

 volc_hapto_hapto <- ggplot(data=res_prim_hapto, aes(x=lfc_std_hapto, y=-log10(p_std_hapto),
col=diff_std_hapto)) + geom_point(size=3) +
   geom_text_repel(aes(label = ifelse(diff_std_hapto, taxon, ""))) +
   theme_classic()+
   labs(x = "Lfc Haptoglobin", y = "-Log10 (p Haptoglobin)") +
   theme(axis.text.x = element_text(size = 16),  # Adjust the size as needed
         axis.text.y = element_text(size = 16))+
   theme(axis.title.x = element_text(size = 16),  # Adjust the size as needed
         axis.title.y = element_text(size = 16))+
   theme(axis.title.x = element_text(margin = margin(t = 13)))+
   theme(axis.title.y = element_text(margin = margin(r = 14)))+
   theme(legend.position = "none")+
   theme(text = element_text(family = "Arial"))
 volc_hapto_hapto

 volc_hapto_cort <- ggplot(data=res_prim_hapto, aes(x=lfc_std_cort, y=-log10(p_std_cort),
col=diff_std_cort)) + geom_point(size=3) +
   geom_text_repel(aes(label = ifelse(diff_std_cort, taxon, ""))) +
   theme_classic()+
   labs(x = "Lfc std. CORT", y = "-Log10 (p std. CORT)") +
   theme(axis.text.x = element_text(size = 16),  # Adjust the size as needed
```

```
         axis.text.y = element_text(size = 16))+
   theme(axis.title.x = element_text(size = 16),  # Adjust the size as needed
         axis.title.y = element_text(size = 16))+
   theme(axis.title.x = element_text(margin = margin(t = 13)))+
   theme(axis.title.y = element_text(margin = margin(r = 14)))+
   theme(legend.position = "none")+
   theme(text = element_text(family = "Arial"))
 volc_hapto_cort

 volc_hapto_age <- ggplot(data=res_prim_hapto, aes(x=lfc_std_age, y=-log10(p_std_age),
col=diff_std_age)) + geom_point(size=3) +
   geom_text_repel(aes(label = ifelse(diff_std_age, taxon, ""))) +
   theme_classic()+
   labs(x = "Lfc std. Age", y = "-Log10 (p std. Age)") +
   theme(axis.text.x = element_text(size = 16),  # Adjust the size as needed
         axis.text.y = element_text(size = 16))+
   theme(axis.title.x = element_text(size = 16),  # Adjust the size as needed
         axis.title.y = element_text(size = 16))+
   theme(axis.title.x = element_text(margin = margin(t = 13)))+
   theme(axis.title.y = element_text(margin = margin(r = 14)))+
   theme(text = element_text(family = "Arial"))
 volc_hapto_age
```

```
# Pseudo-count sensitivity analysis for age

df_age = res_prim_hapto %>% dplyr::select(taxon, ends_with("age")) # create a dataframe with
values only for age

# Sensitivity scores
tab_sens_age <- output_hapto$ss_tab

## Pseudo count sensitivity analysis for age
sens_age <- tab_sens_age %>%
  transmute(taxon, sens_age = std_age) %>%
  left_join(df_age, by = "taxon")
sens_age$diff_std_age = recode(sens_age$diff_std_age * 1,
                               `1` = "Significant",
                               `0` = "Nonsignificant")

fig_sens_age = sens_age %>%
  ggplot(aes(x = taxon, y = sens_age, color = diff_std_age)) +
  geom_point() +
  scale_color_brewer(palette = "Dark2", name = NULL) +
  labs(x = NULL, y = "Sensitivity Score") +
  theme_classic() +
  theme(axis.text.x = element_text(angle = 60, vjust = 0.5))
fig_sens_age
```

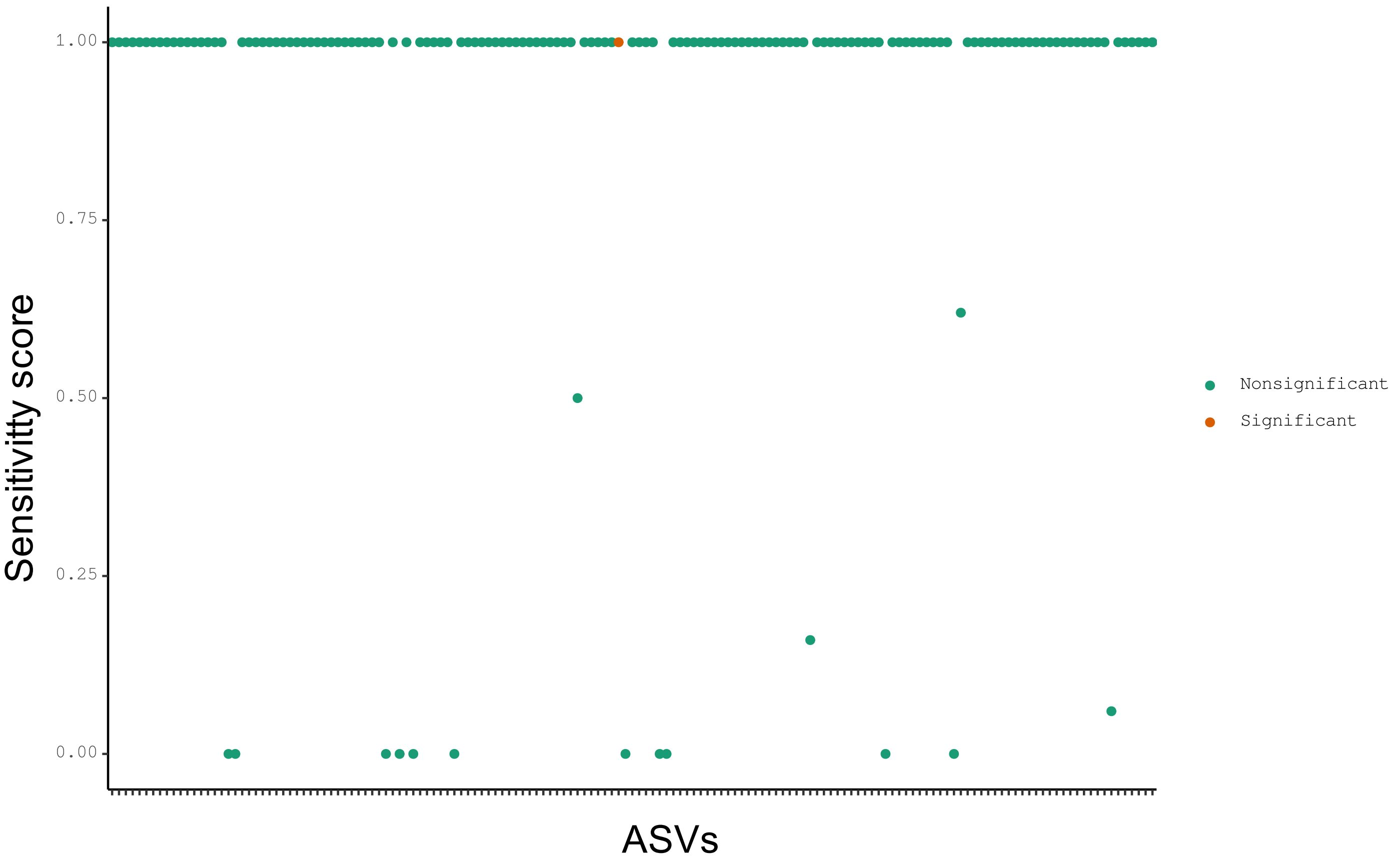

For the co-variate of age, the significant taxa have high sensitivity scores.

B) DAA 28s rRNA

1. Read in the data

```
# read in the phyloseq object
ps_immune <- readRDS("ps_immune.rds")
```

2. Run ANCOM-BC2

2.1 ANCOM-BC2 at ASV level

```
# Diferential abundace analysis on final model.

set.seed(123)
output_immunity = ancombc2(data = ps_immune, assay_name = "counts", tax_level = NULL,
                         fix_formula = "std_bci_two + std_cort + pred_immunity + std_age",
rand_formula = "(1|nest/ring_number)",
                         p_adj_method = "holm", pseudo = 0, pseudo_sens = TRUE,
                         prv_cut = 0.10, lib_cut = 0, s0_perc = 0.05,
                         #group = NULL, struc_zero = TRUE, neg_lb = TRUE,
                         alpha = 0.05, n_cl = 2, verbose = TRUE,
                         #global = TRUE, pairwise = TRUE, dunnet = TRUE, trend = TRUE,
```

```
                         iter_control = list(tol = 1e-2, max_iter = 50, verbose = TRUE),
                         em_control = list(tol = 1e-5, max_iter = 100),
                         lme_control = lme4::lmerControl(optimizer ="Nelder_Mead"),
                         mdfdr_control = list(fwer_ctrl_method = "holm", B = 100),
                         trend_control = list(contrast = list(matrix(c(1, 0, -1, 1),
                         nrow = 2, byrow = TRUE), matrix(c(-1, 0, 1, -1), nrow = 2,
                         byrow = TRUE)), node = list(2, 2), solver = "ECOS", B = 1000))

#run the same model for haptoglobin
output_hapto = ancombc2(data = ps_immune, assay_name = "counts", tax_level = NULL,
                         fix_formula = "std_bci_two + std_cort + std_hapto + std_age",
rand_formula = "(1|nest/ring_number)",
                         p_adj_method = "holm", pseudo = 0, pseudo_sens = TRUE,
                         prv_cut = 0.10, lib_cut = 0, s0_perc = 0.05,
                         #group = NULL, struc_zero = TRUE, neg_lb = TRUE,
                         alpha = 0.05, n_cl = 2, verbose = TRUE,
                         #global = TRUE, pairwise = TRUE, dunnet = TRUE, trend = TRUE,
                         iter_control = list(tol = 1e-2, max_iter = 50, verbose = TRUE),
                         em_control = list(tol = 1e-5, max_iter = 100),
                         lme_control = lme4::lmerControl(optimizer ="Nelder_Mead"),
                         mdfdr_control = list(fwer_ctrl_method = "holm", B = 100),
                         trend_control = list(contrast = list(matrix(c(1, 0, -1, 1),
                         nrow = 2, byrow = TRUE), matrix(c(-1, 0, 1, -1), nrow = 2,
                         byrow = TRUE)), node = list(2, 2), solver = "ECOS", B = 1000))
```

2.1.1 ANCOM-BC2 primary analysis

```
res_prim_immunity = output_immunity$res

res_prim_hapto = output_hapto$res


# Check taxonomy of DAA taxa

DAA_bci <- c("ASV_201")
DAA_bci_ps <- prune_taxa(taxa_names(ps_immune) %in% DAA_bci, ps_immune)
DAA_bci_ps@tax_table

            #Kingdom        Phylum       Class              Order          Family         Genus
      Species
#ASV_201  "d__Eukaryota" "Ascomycota" "Dothideomycetes" "Dothideales" "Dothideales"
"Dothideales" "Hormonema_carpetanum"

DAA_age <- c("ASV_381")
DAA_age_ps <- prune_taxa(taxa_names(ps_immune) %in% DAA_age, ps_immune)
DAA_age_ps@tax_table

             #Kingdom        Phylum               Class              Order
Family               Genus            Species
#ASV_381"d__Eukaryota;p_Phragmoplastophyta;c_Phragmoplastophyta;o_Phragmoplastophyta;f_Phragmop
lastophyta;g_Phragmoplastophyta;s_Pinus_taeda"
```

Two deferentially abundant ASVs co-vary with Age and BCI when the model incorporates the variable immunity.
ASV that co-varies with age did not pass sensitivity analysis
No differential abundant taxa found when modelling haptoglobin

2.1.2 Sensitivity scores

```
tab_sens_immunity = output_immunity$ss_tab

tab_sens_hapto = output_hapto$ss_tab
```

2.2.3 Plot ANCOM-BC2 results

```
# Volcano plots - immunity
volc_immunity_bci <- ggplot(data=res_prim_immunity, aes(x=lfc_std_bci_two, y=-
log10(p_std_bci_two), col=diff_std_bci_two)) + geom_point(size=3) +
   geom_text_repel(aes(label = ifelse(diff_std_bci_two, taxon, ""))) +
   theme_classic()+
  labs(x = "Lfc std. BCI", y = "-Log10 (p std. BCI)") +
  theme(axis.text.x = element_text(size = 16),  # Adjust the size as needed
        axis.text.y = element_text(size = 16))+
  theme(axis.title.x = element_text(size = 16),  # Adjust the size as needed
        axis.title.y = element_text(size = 16))+
  theme(axis.title.x = element_text(margin = margin(t = 13)))+
  theme(axis.title.y = element_text(margin = margin(r = 14)))+
  theme(legend.position = "none")+
  theme(text = element_text(family = "Arial"))
 volc_immunity_bci

 volc_immunity_immunity <- ggplot(data=res_prim_immunity, aes(x=lfc_pred_immunity, y=-
log10(p_pred_immunity), col=diff_pred_immunity)) + geom_point(size=3) +
   geom_text_repel(aes(label = ifelse(diff_pred_immunity, taxon, ""))) +
   theme_classic()+
   labs(x = "Lfc Immunity", y = "-Log10 (p Immunity)") +
   theme(axis.text.x = element_text(size = 16),  # Adjust the size as needed
         axis.text.y = element_text(size = 16))+
   theme(axis.title.x = element_text(size = 16),  # Adjust the size as needed
         axis.title.y = element_text(size = 16))+
   theme(axis.title.x = element_text(margin = margin(t = 13)))+
   theme(axis.title.y = element_text(margin = margin(r = 14)))+
   theme(legend.position = "none")+
   theme(text = element_text(family = "Arial"))
 volc_immunity_immunity

 volc_immunity_cort <- ggplot(data=res_prim_immunity, aes(x=lfc_std_cort, y=-log10(p_std_cort),
col=diff_std_cort)) + geom_point(size=3) +
   geom_text_repel(aes(label = ifelse(diff_std_cort, taxon, ""))) +
   theme_classic()+
   labs(x = "Lfc std. CORT", y = "-Log10 (p std. CORT)") +
   theme(axis.text.x = element_text(size = 16),  # Adjust the size as needed
         axis.text.y = element_text(size = 16))+
   theme(axis.title.x = element_text(size = 16),  # Adjust the size as needed
         axis.title.y = element_text(size = 16))+
   theme(axis.title.x = element_text(margin = margin(t = 13)))+
   theme(axis.title.y = element_text(margin = margin(r = 14)))+
   theme(legend.position = "none")+
   theme(text = element_text(family = "Arial"))
 volc_immunity_cort

 volc_immunity_age <- ggplot(data=res_prim_immunity, aes(x=lfc_std_age, y=-log10(p_std_age),
col=diff_std_age)) + geom_point(size=3) +
   geom_text_repel(aes(label = ifelse(diff_std_age, taxon, ""))) +
```

```
   theme_classic()+
   labs(x = "Lfc std. Age", y = "-Log10 (p std. Age)") +
   theme(axis.text.x = element_text(size = 16),  # Adjust the size as needed
         axis.text.y = element_text(size = 16))+
   theme(axis.title.x = element_text(size = 16),  # Adjust the size as needed
         axis.title.y = element_text(size = 16))+
   theme(axis.title.x = element_text(margin = margin(t = 13)))+
   theme(axis.title.y = element_text(margin = margin(r = 14)))+
   theme(text = element_text(family = "Arial"))
 volc_immunity_age


# Volcano plots - haptoglobin

volc_hapto_bci <- ggplot(data=res_prim_hapto, aes(x=lfc_std_bci_two, y=-log10(p_std_bci_two),
col=diff_std_bci_two)) + geom_point(size=3) +
   geom_text_repel(aes(label = ifelse(diff_std_bci_two, taxon, ""))) +
   theme_classic()+
  labs(x = "Lfc std. BCI", y = "-Log10 (p std. BCI)") +
  theme(axis.text.x = element_text(size = 16),  # Adjust the size as needed
        axis.text.y = element_text(size = 16))+
  theme(axis.title.x = element_text(size = 16),  # Adjust the size as needed
        axis.title.y = element_text(size = 16))+
  theme(axis.title.x = element_text(margin = margin(t = 13)))+
  theme(axis.title.y = element_text(margin = margin(r = 14)))+
  theme(legend.position = "none")+
  theme(text = element_text(family = "Arial"))
 volc_hapto_bci

 volc_hapto_hapto <- ggplot(data=res_prim_hapto, aes(x=lfc_std_hapto, y=-log10(p_std_hapto),
col=diff_std_hapto)) + geom_point(size=3) +
   geom_text_repel(aes(label = ifelse(diff_std_hapto, taxon, ""))) +
   theme_classic()+
   labs(x = "Lfc Haptoglobin", y = "-Log10 (p Haptoglobin)") +
   theme(axis.text.x = element_text(size = 16),  # Adjust the size as needed
         axis.text.y = element_text(size = 16))+
   theme(axis.title.x = element_text(size = 16),  # Adjust the size as needed
         axis.title.y = element_text(size = 16))+
   theme(axis.title.x = element_text(margin = margin(t = 13)))+
   theme(axis.title.y = element_text(margin = margin(r = 14)))+
   theme(legend.position = "none")+
   theme(text = element_text(family = "Arial"))
 volc_hapto_hapto

 volc_hapto_cort <- ggplot(data=res_prim_hapto, aes(x=lfc_std_cort, y=-log10(p_std_cort),
col=diff_std_cort)) + geom_point(size=3) +
   geom_text_repel(aes(label = ifelse(diff_std_cort, taxon, ""))) +
   theme_classic()+
   labs(x = "Lfc std. CORT", y = "-Log10 (p std. CORT)") +
   theme(axis.text.x = element_text(size = 16),  # Adjust the size as needed
         axis.text.y = element_text(size = 16))+
   theme(axis.title.x = element_text(size = 16),  # Adjust the size as needed
         axis.title.y = element_text(size = 16))+
   theme(axis.title.x = element_text(margin = margin(t = 13)))+
   theme(axis.title.y = element_text(margin = margin(r = 14)))+
   theme(legend.position = "none")+
   theme(text = element_text(family = "Arial"))
 volc_hapto_cort
```

```
 volc_hapto_age <- ggplot(data=res_prim_hapto, aes(x=lfc_std_age, y=-log10(p_std_age),
col=diff_std_age)) + geom_point(size=3) +
   geom_text_repel(aes(label = ifelse(diff_std_age, taxon, ""))) +
   theme_classic()+
   labs(x = "Lfc std. Age", y = "-Log10 (p std. Age)") +
   theme(axis.text.x = element_text(size = 16),  # Adjust the size as needed
         axis.text.y = element_text(size = 16))+
   theme(axis.title.x = element_text(size = 16),  # Adjust the size as needed
         axis.title.y = element_text(size = 16))+
   theme(axis.title.x = element_text(margin = margin(t = 13)))+
   theme(axis.title.y = element_text(margin = margin(r = 14)))+
   theme(text = element_text(family = "Arial"))
 volc_hapto_age
```

```
# Pseudo count sensitivity analysis

df_bci_asv = res_prim_immunity %>% dplyr::select(taxon, ends_with("two")) # create a dataframe
with values only for bci

df_age_asv = res_prim_immunity %>% dplyr::select(taxon, ends_with("age")) # create a dataframe
with values only for age

# Pseudo count sensitivity analysis for BCI
sens_asv_bci = tab_sens_immunity %>%
  transmute(taxon, sens_asv_bci = std_bci_two) %>%
  left_join(df_bci_asv, by = "taxon")

sens_asv_bci$diff_std_bci_asv = recode(sens_asv_bci$diff_std_bci_two * 1,
                                       `1` = "Significant",
                                       `0` = "Non-significant")

fig_sens_asv_bci = sens_asv_bci %>%
  ggplot(aes(x = taxon, y = sens_asv_bci, color = diff_std_bci_two)) +
  geom_point() +
  scale_color_brewer(palette = "Dark2", name = NULL) +
  labs(x = "ASVs", y = "Sensitivity Score") +
  theme_bw()

fig_sens_asv_bci

# Pseudo count sensitivity analysis for Age
sens_asv_age = tab_sens_immunity %>%
  transmute(taxon, sens_asv_age = std_age) %>%
  left_join(df_age_asv, by = "taxon")

sens_asv_bci$diff_std_age_asv = recode(sens_asv_age$diff_std_age * 1,
                                       `1` = "Significant",
                                       `0` = "Non-significant")

fig_sens_asv_age = sens_asv_age %>%
  ggplot(aes(x = taxon, y = sens_asv_age, color = diff_std_age)) +
  geom_point() +
  scale_color_brewer(palette = "Dark2", name = NULL) +
  labs(x = "ASVs", y = "Sensitivity Score") +
  theme_bw()
```

```
fig_sens_asv_age
```

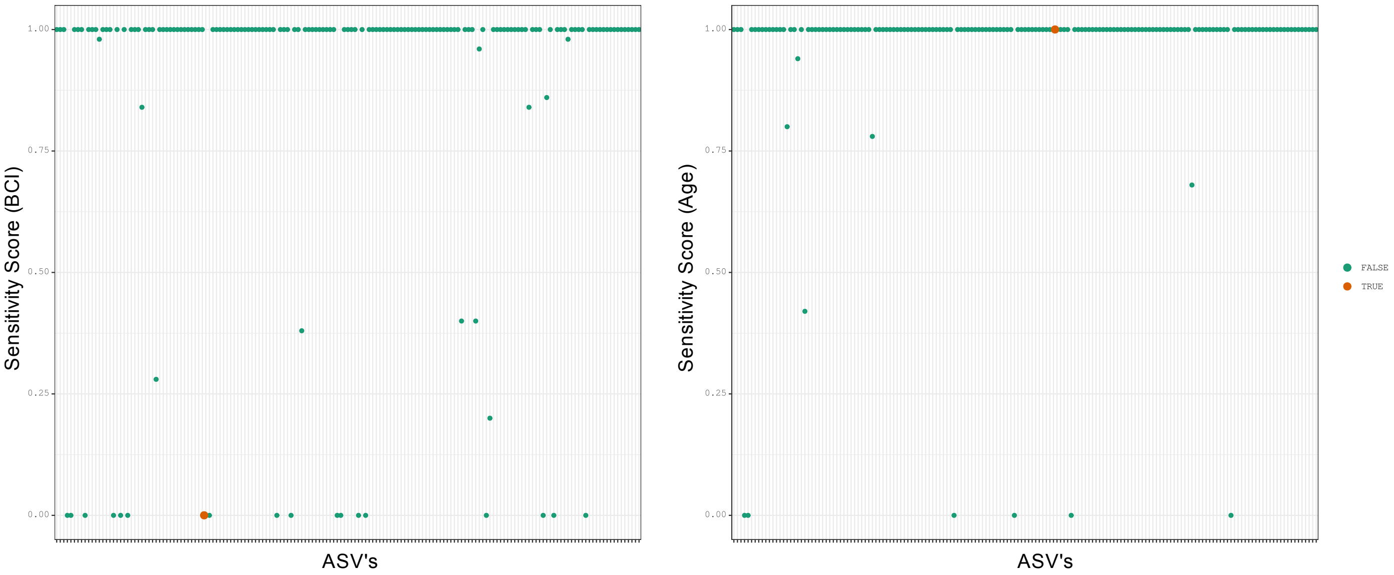

For the co-variate of Age the deferentially abundant ASV has high sensitivity scores

```
# Plot log fold changes with unit of BCI
df_fig_bci_asv = df_bci_asv %>%
  filter(diff_std_bci_two == TRUE) %>%
  arrange(desc(lfc_std_bci_two)) %>%
  mutate(direct = ifelse(lfc_std_bci_two > 0, "Positive LFC", "Negative LFC")) # prepare the
dataframe for plotting

df_fig_bci_asv$taxon = factor(df_fig_bci_asv$taxon, levels = df_fig_bci_asv$taxon)
df_fig_bci_asv$direct = factor(df_fig_bci_asv$direct,
                               levels = c("Positive LFC", "Negative LFC"))

fig_bci_asv = df_fig_bci_asv %>%
  ggplot(aes(x = taxon, y = lfc_std_bci_two, fill = direct)) +
  geom_bar(stat = "identity", width = 0.7, color = "black",
           position = position_dodge(width = 0.4)) +
  geom_errorbar(aes(ymin = lfc_std_bci_two - se_std_bci_two, ymax = lfc_std_bci_two +
se_std_bci_two),
                width = 0.2, position = position_dodge(0.05), color = "black") +
  labs(x = NULL, y = "Log fold change", title = "Log fold changes as one unit increase of BCI")
+
  scale_fill_discrete(name = NULL) +
  scale_color_discrete(name = NULL) +
  theme_bw() +
  theme(plot.title = element_text(hjust = 0.5),
        panel.grid.minor.y = element_blank(),
        axis.text.x = element_text(angle = 60, hjust = 1))
fig_bci_asv
```

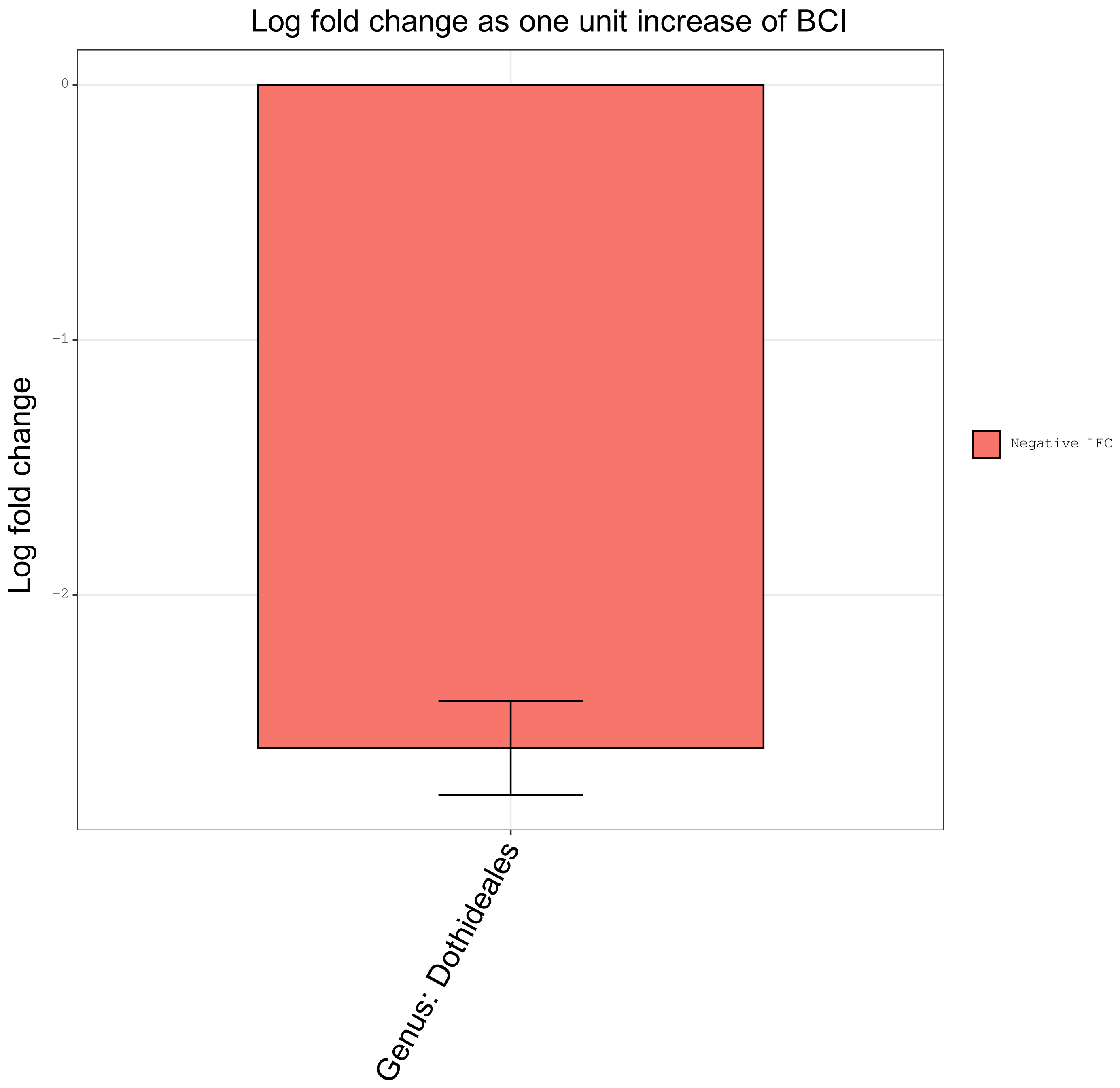
Log fold change as one unit increase of BCI
0
−1
−2
Log fold change
Negative LFC
Genus: Dothideales
